# Quantum and thermodynamic evaluation of C_24_ fullerene-based nanosensors for detection of mydayis in biomedical and drug detection applications

**DOI:** 10.1038/s41598-025-34744-3

**Published:** 2026-01-06

**Authors:** Mohammed Ghazwani, Umme Hani

**Affiliations:** Department of Pharmaceutics, College of Pharmacy, King Khalid University, 62223 Al Faraa, Abha, Saudi Arabia

**Keywords:** Mydayis, Nanosensors, Adsorption, Drug detection, DFT, Chemistry, Materials science, Nanoscience and technology, Physics

## Abstract

From a public health and forensic perspective, the detection of Mydayis (a long-acting amphetamine-based drug) is crucial due to its psychotropic effects. In this work, Density Functional Theory (DFT), Time-Dependent DFT (TD-DFT), and Quantum Theory of Atoms in Molecules (QTAIM) were employed to evaluate pristine C_24_ fullerene and its doped derivatives (BC_23_ and SiC_23_) as nanosensors for Mydayis. Key electronic, thermodynamic, optical, and adsorption-based parameters were calculated, and the IR spectrum simulated using DFT showed strong agreement with experimental IR data reported in the literature, confirming the reliability of the computational approach. Among the investigated structures, pristine C24 was identified as the most effective disposable electrochemical sensor, exhibiting moderate adsorption energy (− 23.88 kcal.mol^−1^), a measurable conductivity increase (2.74 × 10^9^ → 2.77 × 10^9^ A.m^−2^), and significant enhancements in dipole moment (0.00 → 11.253 D) and polarizability (170.8 → 285.994 a.u.). In contrast, BC_23_ and SiC_23_ demonstrated exceptionally strong adsorption behavior (− 53.09 and − 54.00 kcal.mol^−1^, respectively) and extremely long recovery times (8.13 × 10^26^ and 3.80 × 10^27^ s), establishing them as excellent high-capacity absorbers for irreversible Mydayis capture. Additionally, BC_23_ exhibited the most pronounced colorimetric response, with a dramatic bathochromic shift from 432 to 655 nm upon Mydayis binding, confirming its role as the best disposable colorimetric sensor. These findings highlight the complementary roles of C_24_, BC_23_, and SiC_23_ in the electrochemical, optical, and adsorptive detection of Mydayis and provide a robust theoretical foundation for future experimental sensor development.

**Supplementary Information:**

The online version contains supplementary material available at 10.1038/s41598-025-34744-3.

## Introduction

Identifying narcotics is crucial for several reasons, including public health, law enforcement, and medical safety^1–3^. Among drugs, the identification of Mydayis is of particular importance due to its potential for use as a narcotic. Mydayis, a long-acting mixed amphetamine salt formulation, poses significant health risks when taken without appropriate medical oversight or in doses higher than prescribed. Because it remains active in the body for up to sixteen hours, it can lead to sustained elevations in heart rate and blood pressure, placing strain on the cardiovascular system and increasing the likelihood of arrhythmias, chest pain, or, in extreme cases, cardiac events^4,5^. Prolonged stimulation of the central nervous system can cause agitation, anxiety, insomnia, and, at higher exposures, hallucinations or psychotic symptoms. The extended duration of action also raises the risk of sleep deprivation, which can impair judgment, attention, and emotional stability. Over time, the brain’s response to dopamine and norepinephrine signaling may become altered, resulting in dependence or withdrawal symptoms when the drug is stopped. Identifying Mydayis in clinical, forensic, or educational settings is crucial because its appearance and pharmacology closely resemble other amphetamine medications, making accidental ingestion or unrecognized exposure possible^6^. Early recognition allows for appropriate medical intervention, prevents potential cardiovascular or neurological harm, and helps ensure that any observed behavioral or physiological changes are correctly attributed to the drug’s prolonged stimulant effect rather than to another underlying cause^7,8^.

The detection of Mydayis can be performed using various methods, including chromatography (e.g., HPLC, GC–MS), immunoassays, and spectroscopy (e.g., UV–Vis, FTIR). While these techniques are reliable, they suffer from significant drawbacks, such as the need for expensive equipment, time-consuming sample preparation, and the requirement for skilled personnel. Additionally, some methods lack portability, making them unsuitable for rapid on-site testing. Due to these limitations, there is a growing demand for fast, cost-effective, and accessible detection methods that can be deployed in various settings, from clinical to law enforcement applications^9–11^.

Recently, electrochemical and colorimetric sensors have emerged as promising alternatives due to their simplicity, high sensitivity, and potential for miniaturization^12^. These sensors leverage nanomaterials to enhance detection performance, with carbon nanomaterials (such as graphene, carbon nanotubes, and fullerenes) playing a key role in their development^13^. Among these sensors, fullerenes, and in particular C_24_ fullerene, have attracted attention due to their unique electronic and structural properties, making them excellent candidates for sensor design. The C_24_ fullerene represents one of the smallest stable closed-cage carbon clusters, consisting of 24 carbon atoms arranged in a highly curved polyhedral geometry. Several previous theoretical and experimental reports on low-order fullerenes and heteroatom-substituted carbon clusters have supported the synthetic accessibility of pristine C_24_ and its doped derivatives. Although the direct isolation of C_24_ remains experimentally challenging due to its small cage size, fragment-based and laser-ablation methods have successfully generated analogous carbon clusters. These findings generally indicate that achieving both pristine and doped C_24_ structures is, in principle, synthetically achievable^14^. Also, unlike larger fullerenes such as C_60_ and C_70_, C_24_ possesses a greater degree of surface curvature and higher strain energy, resulting in increased chemical reactivity and stronger orbital overlap with adsorbates. The smaller cage size leads to a larger surface-to-volume ratio and a higher density of localized π-electrons at the pentagonal and hexagonal junctions, which facilitates enhanced charge transfer during adsorption. Quantum chemical analyses have shown that the frontier molecular orbitals of C_24_ are more polarized and energetically accessible than those of C_60_, enabling more efficient electron donation or acceptance depending on the interacting species. Additionally, its relatively low cohesive energy and high dipole polarizability make it an excellent platform for electronic perturbation under external fields (an essential attribute for sensor design). Additionally, its distinct topology results in a larger HOMO–LUMO gap and an enhanced local charge density compared to larger fullerenes, such as C_60_, making it more chemically active in adsorption and charge transfer processes. Specifically, C24’s paramount advantage is its exceptionally high electron affinity. While C_60_ is already renowned for being a good electron acceptor (a property crucial for its use in organic photovoltaics) C_24_ possesses a substantially higher electron affinity^15,16^. Also, Jana et al. recently emphasized the role of C_24_ in the recognition of drugs such as amphetamine, ketamine and mercaptopurine^17^.

Recent studies have also highlighted the potential of C_24_ fullerene in sensor applications. For instance, Huwaimel et al. demonstrated its effectiveness in detecting gamma-butyrolactone, while Tayebi-Moghaddam et al. explored its use for acrylamide detection^18,19^. Additionally, Baei et al. showed its sensitivity toward nicotine molecules^20^. Furthermore, research has indicated that doping C_24_ fullerene with elements such as boron (B) and silicon (Si) can enhance its electronic and quantum properties, further optimizing its sensing capabilities^21,22^. Boron (B) doping creates an electron deficiency and improves interactions with electron-rich molecules, while silicon (Si) increases surface reactivity and electrical conductivity^23,24^. Therefore, functionalization of C_24_ fullerenes with these metals is expected to allow for more efficient detection. Although based on this evidence, it is predicted that doping these atoms will cause significant changes in the electronic properties of C_24_, the effect of doping other atoms can be useful in improving the electronic properties of C_24_ and should definitely be considered in future studies.

Also, given the limitations of conventional laboratory techniques, computational chemistry and physics methods (such as Density Functional Theory (DFT), Time-Dependent DFT (TD-DFT), and Quantum Theory of Atoms in Molecules (QTAIM)) have become invaluable tools for predicting sensor performance before experimental synthesis. These methods reduce costs, save time, and provide deep insights into molecular interactions at the atomic level^25–27^.

In this research, we investigate the potential of C_24_ fullerene and its doped forms (B, and Si) as electrochemical/colorimetric sensors for Mydayis detection using DFT, TD-DFT, and QTAIM theories. By analyzing electronic properties, adsorption mechanisms, and charge transfer behavior, we aim to identify the most efficient sensor configuration. We believe this study will contribute to the development of a new generation of highly sensitive and selective sensors, guiding experimentalists in synthesizing advanced materials for real-world Mydayis detection applications.

## Computational details

**Fig. 1:**
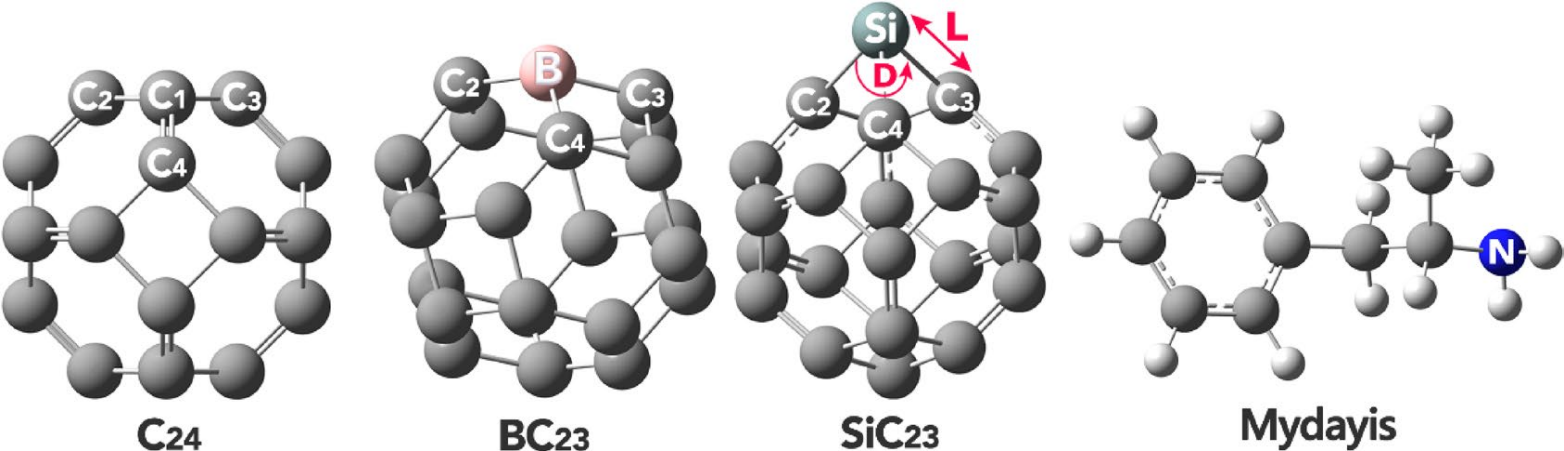
Optimized geometry of each of the studied structures.

All the designed structures, including C_24_, BC_23_, SiC_23_, and the drug molecule Mydayis, were initially modeled using GaussView 6.0 software to construct their molecular geometries (Fig. 1) (The XYZ coordinates of all structures were reported in the Supplementary Data (Table S1).)^28^. Following this, geometric optimizations were carried out using the Gaussian 09W computational package^29^. The optimizations were performed at the WB97XD/6-31G(d) level of theory. The WB97XD functional was selected due to its hybrid nature and its incorporation of empirical dispersion corrections (denoted by “XD”), which are essential for accurately modeling non-covalent interactions, such as π–π stacking, van der Waals forces, and hydrogen bonding^30^.

It is important to note that Mydayis, as an amphetamine derivative, may exist in protonated form under physiological pH conditions. However, in this study, the neutral (non-protonated) form was used for all simulations. This approach was chosen to maintain direct comparability with previous computational works on drug adsorption to carbon nanostructures and to focus on intrinsic electronic and thermodynamic interaction mechanisms independent of charge effects. All optimizations were conducted in the water phase to mimic physiological conditions, employing the Conductor-like Polarizable Continuum Model (CPCM) to account for solvation effects. The CPCM model partially accounts for electrostatic screening associated with protonation in aqueous environments. Future work will extend this analysis to the protonated form to further elucidate pH-dependent interaction behavior^31^. Frequency calculations at the same theoretical level confirmed the stability of all structures, as evidenced by the absence of imaginary frequencies, ensuring that the optimized geometries represent true energy minima rather than transition states.

To investigate the electronic excitation properties and potential colorimetric detection capabilities, time-dependent DFT (TD-DFT) calculations were performed on each structure (both in the presence and absence of Mydayis) to simulate their UV–Vis adsorption spectra. This analysis helps identify key spectral shifts that could serve as optical signatures for Mydayis detection^26^.

Furthermore, the Quantum Theory of Atoms in Molecules (QTAIM) was applied to characterize the nature of intermolecular interactions at the bond critical points (BCPs) between C_24_ fullerene (and its doped forms) and Mydayis. This approach provides critical insights into the strength and type of bonding (e.g., covalent, electrostatic, or van der Waals), which are essential for understanding the sensing mechanism at the atomic level^32^.

The cohesive energy (E_Coh_) quantifies the average binding strength between atoms in the nanostructure, serving as a measure of its structural stability. It was calculated according to Eq. (1):

1 $$E_{Coh} = - \left( {E_{total} - \mathop \sum \limits_{i} n_{i} E_{i} } \right)/n.$$

where Etotal is the total energy of the optimized structure (C_24_ or its doped analogs BC_23_, and SiC_23_), Ei: is the total energy of an optimized isolated atom of type i (C, B, or Si), ni is the number of atoms of that i type, and n is the total number of atoms in the system. A larger cohesive energy implies stronger atomic bonding and greater thermodynamic stability of the structure^33^.

The energy gap (HLG), chemical softness (S), chemical hardness ($\upeta$), and chemical potential (μ) were calculated using Eqs. 2–5^34^.

2 $$\mathrm{HLG}=\left|{\mathrm{E}}_{\mathrm{HOMO}}-{\mathrm{E}}_{\mathrm{LUMO}}\right|$$

3 $$\upeta =\raisebox{1ex}{$(-{\mathrm{E}}_{\mathrm{HOMO}}-(-{\mathrm{E}}_{\mathrm{LUMO}} ))$}\!\left/ \!\raisebox{-1ex}{$2$}\right.$$

4 $$\upmu =-(-{\mathrm{E}}_{\mathrm{HOMO}}+(-{\mathrm{E}}_{\mathrm{LUMO}}))/2$$

5 $$S=1/2\upeta$$

E_HOMO_ = Energy of highest occupied molecular orbital (HOMO), E_LUMO_ = Energy of lowest unoccupied molecular orbital (LUMO).

The maximum charge transferred (ΔNmax) and charge transfer based on electrophilicity (ECT) were calculated using Eqs. 6 and 7, respectively.

6 $${\Delta N}_{max}=-\raisebox{1ex}{$\mu $}\!\left/ \!\raisebox{-1ex}{$\eta $}\right.$$

7 $$ECT={({\Delta N}_{max})}_{\alpha }-{\left({\Delta N}_{max}\right)}_{\beta }$$

${({\Delta N}_{max})}_{\alpha }$ represents the maximum charge transferred by the complex and represents the maximum charge transferred by the sensor. A positive ECT value indicates no net electron transfer from the sensor to Mydayis (the sensor acts as an electron donor). In contrast, a negative ECT value indicates electron transfer from Mydayis to the sensor (the sensor acts as an electron acceptor)^35^.

Recovery time (τ) and electrical conductivity ($\upsigma$) were calculated using Eqs. 8 and 9, respectively.

8 $$\tau ={V}_{0}^{-1}\times \mathrm{exp}(-\frac{{E}_{ads}}{{k}_{B}T})$$

9 $$\upsigma =A{T}^{3/2}{e}^{(-Eg/2KT)}$$

In these equations: A = Richardson constant (6 × 10^5^ A.m^−2^), k_B_ = Boltzmann constant, E_ads_ = adsorption energy, V_0_ = attempt frequency (10^12^ s^-1^), and, T = temperature (298 K)^36^.

The adsorption energy (E_ads_), enthalpy change (ΔH_ads_), and Gibbs free energy change (ΔG_ads_) were calculated for each complex, respectively, using Eq. 10–12.

10 $${E}_{ads}={E}_{\left(R-\right)C24@Mydayis}-\left({E}_{\mathrm{Mydayis}}+{E}_{\left(R-\right)C24}\right)+{E}_{BSSE}$$

11 $${\Delta H}_{ads}={H}_{\left(R-\right)C24@Mydayis}-\left({H}_{Mydayis}+{H}_{\left(R-\right)C24}\right)$$

12 $${\Delta G}_{ads}={G}_{\left(R-\right)C24@Mydayis}-\left({G}_{Mydayis}+{G}_{\left(R-\right)C24}\right)$$

In these equations: E_(R-)C24@Mydayis_ = Total energy of the optimized complex (Mydayis adsorbed on R-functionalized C_23_, where R = B, Si,or pure C_24_), E_Mydayis_ = Energy of the isolated Mydayis molecule (optimized geometry), E_(R-)C24_ = Energy of the isolated functionalized C_24_ fullerene (R = B/Si or pure C_23_) in its optimized form, and E_BSSE_ = Basis Set Superposition Error correction^37^.

The dipole moment and polarizability were calculated for each of the structures using Eqs. 13 and 14^38^.

13 $$\mu =\sqrt{{\mu }_{x}^{2}+{\mu }_{y}^{2}+{\mu }_{z}^{2}}$$

14 $$\alpha =\frac{1}{3}\left({\alpha }_{xx}+{\alpha }_{yy}+{\alpha }_{zz}\right)$$

Collectively, these computational strategies ensure a comprehensive evaluation of C_24_ fullerene’s potential as an electrochemical/colorimetric sensor for Mydayis, bridging theoretical predictions with practical sensing applications.

## Structural properties

### Structural properties

#### Bond length and bond angle

The precise analysis of bond lengths and bond angles is fundamental in the design and optimization of molecular structures, as these geometric parameters directly influence the electronic properties, stability, and reactivity of a molecule. At the atomic level, even minor deviations in bond lengths or angles can significantly alter the distribution of electron density, particularly in conjugated systems where π-electron delocalization plays a crucial role. For instance, in carbon-based nanomaterials like C_24_ fullerene, changes in bond lengths can modify π-orbital overlap, thereby affecting charge transport, optical properties, and intermolecular interactions^39^. In this regard, the bond length and bond angles for each of the designed structures were computationally studied and the results were reported in Table 1.

**Table 1:** Bond length (in Angstroms (Å)) and bond angle (in Degrees (^o^)) values in each of the devices studied.

Structure	L (Å)		D (^o^)	
C_24_	C2-C1	1.48	C2-C1-C3	90.02
	C3-C1	1.48	C2-C1-C4	120.04
	C4-C1	1.36	C3-C1-C4	120.00
BC_23_	C2-B	1.56	C2-B-C3	120.16
	C3-B	1.54	C2-B-C4	89.78
	C4-B	1.56	C3-B-C4	120.17
SiC_23_	C2-Si	1.94	C2-Si-C3	68.56
	C3-Si	1.94	C2-Si-C4	87.94
	C4-Si	1.91	C3-Si-C4	87.94

The analysis of bond lengths and angles in Table 1 reveals crucial insights into how structural modifications affect π-electron mobility in C_24_ fullerene and its doped derivatives (BC_23_, SiC_23_), which directly influences their potential as molecular sensors. In pristine C_24_ fullerene, the balanced bond lengths (C2–C1: 1.48 Å, C4-C1: 1.36 Å) and near-ideal bond angles (≈120°) create an optimal conjugated system for π-electron delocalization, essential for efficient charge transport in sensing applications. However, doping introduces significant structural changes that alter this π-conjugation network. Boron doping (BC_23_) maintains reasonable sp2 hybridization (≈ 120°) but introduces longer bonds (1.54–1.56 Å) that moderately disrupt π–electron uniformity while potentially enhancing charge transfer through electron deficiency. The silicon-doped structure (SiC_23_) exhibits more dramatic effects, with substantially elongated bonds (1.84–1.94 Å) and severely compressed angles (68.56–71.56°) that significantly impair π-electron mobility due to poor orbital overlap and a transition toward sp3 hybridization. These structural modifications demonstrate a clear trade-off between introducing dopant-specific functionalities and maintaining the π-conjugated framework necessary for effective sensing. The findings highlight that while doping can tailor electronic properties for specific applications, excessive structural distortion from certain dopants may compromise the π-electron delocalization critical for sensor performance, suggesting that careful selection of dopants is essential for optimizing these nanomaterials for drug detection.

#### Cohesive energy

Cohesive energy represents the binding strength of atoms within a material, quantifying the energy required to separate them into individual components. Examining cohesive energy after doping is crucial because it reveals how the introduction of foreign atoms affects the structural stability and integrity of the host material (such as C_24_ fullerene). A significant decrease in cohesive energy post-doping may indicate weakened atomic bonds and potential structural instability, while maintained or enhanced cohesive energy suggests the doped system remains robust. This analysis helps predict the material’s durability under operational conditions. It ensures that the doping process has not compromised the fundamental stability needed for reliable sensor performance in pharmaceutical or biomedical applications^40^.

**Fig. 2:**
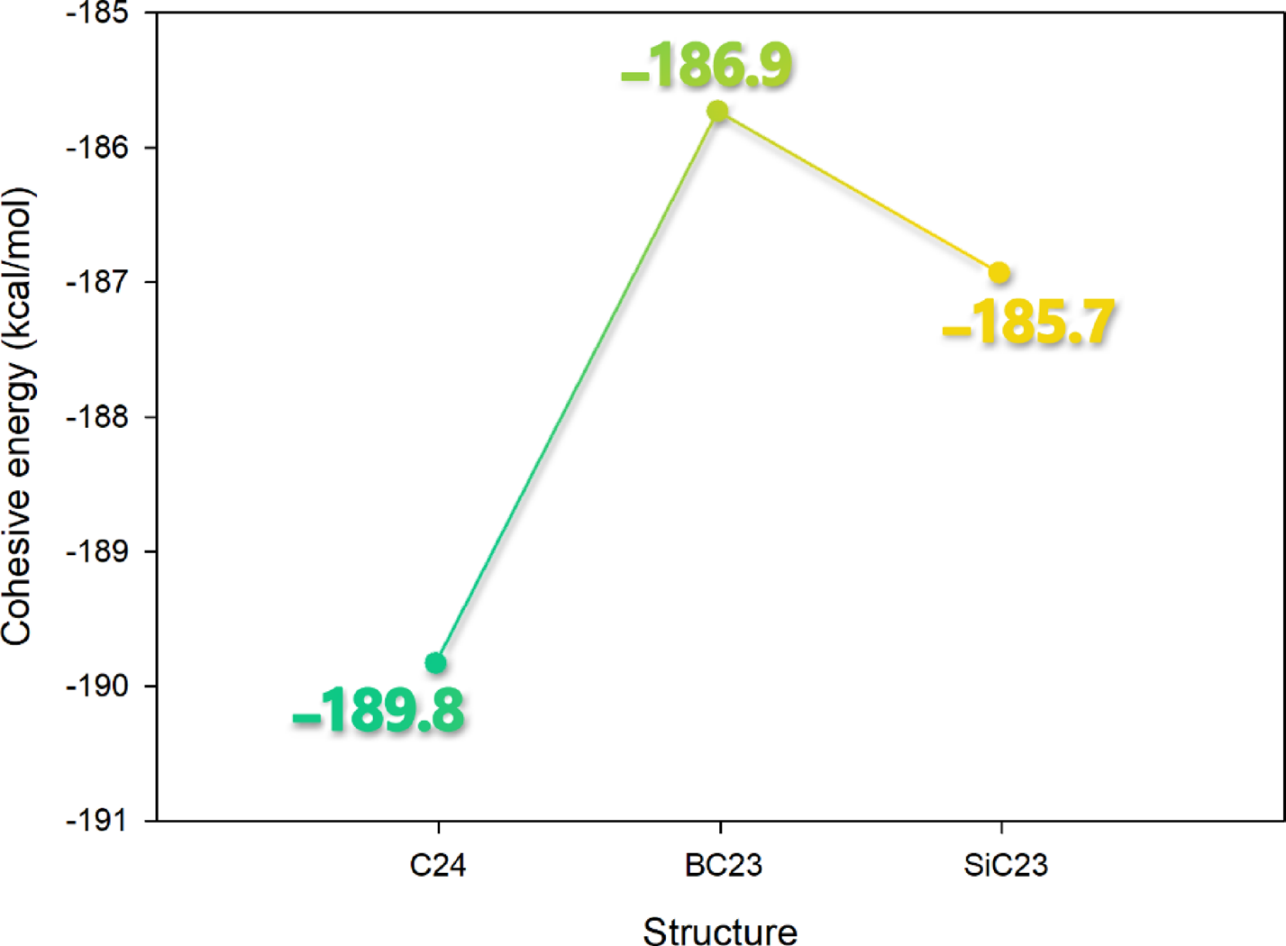
Cohesive energy change trend after doping fullerenes-C_24_ with B, O, P, and Si.

The cohesive energy data reveal interesting variations among the different structures, with the pristine C_24_ fullerene exhibiting the highest cohesive energy (− 189.8 kcal.mol^−1^), followed by BC_23_ (− 186.9 kcal.mol^−1^) and SiC_23_ (− 185.7 kcal.mol⁻^1^) (Fig. 2). The relatively small differences between the doped structures suggest that the various dopants (B, Si) have similar impacts on the overall structural stability of the fullerene framework. The boron-doped system (BC_23_) shows the least reduction in cohesive energy compared to pristine C_24_ (1.5% decrease). These minor variations likely reflect differences in atomic sizes, electronegativities, and bonding characteristics of the dopant elements, with boron’s intermediate properties appearing to best preserve the original bonding network. The data generally show that while all impurities affect fullerene stability to some extent, none cause a significant change.

#### IR spectrum

Examining IR spectra is crucial in molecular design because it provides direct information about the functional groups, bond types, and structural features of a molecule. These spectra help confirm the existence and integrity of key chemical bonds, detecting structural changes^41^.

**Fig. 3:**
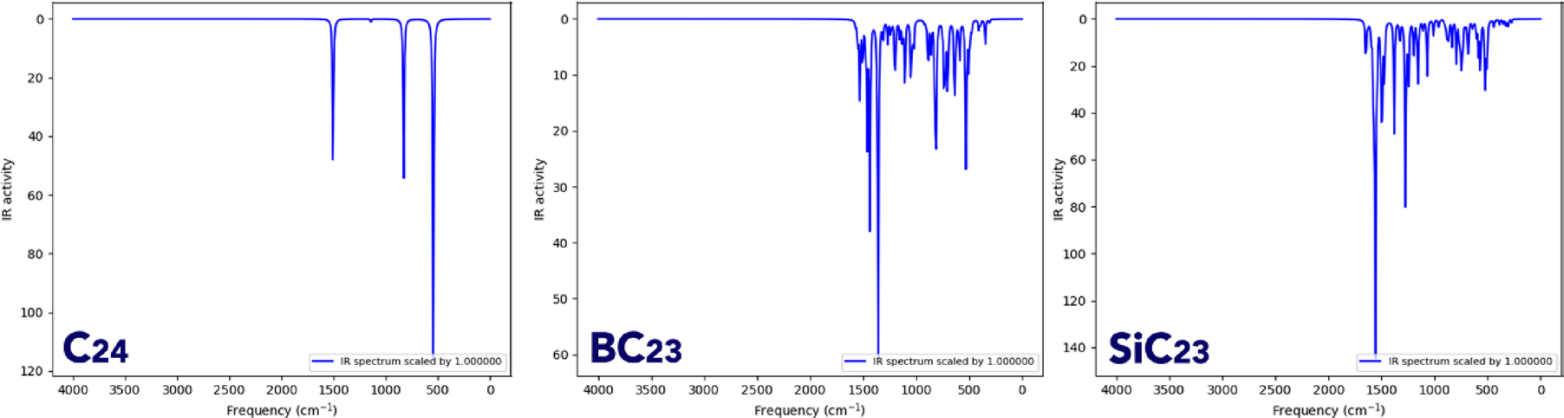
IR spectrum obtained for pristine C_24_ fullerene.

**Fig. 4:**
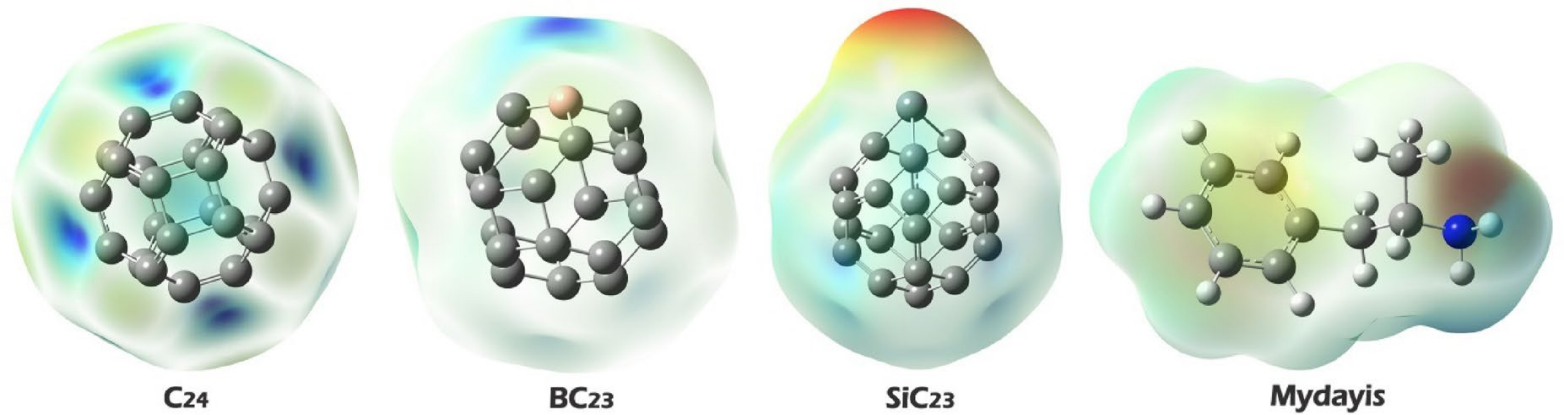
MEP map for Mydayis, Prestin C_24_, and its doping forms.

The infrared (IR) spectra of the pristine C_24_ cluster and its heteroatom‑substituted derivatives (BC_23_ and SiC_23_) demonstrate the pronounced influence of boron, oxygen, silicon, and phosphorus doping on vibrational activity in carbon nanostructures (Figs. 3 and 4).

As expected, C_24_ (a symmetric, pure carbon cluster) exhibits a relatively sparse IR spectrum, characterized by a few sharp peaks in the 1600–600 cm^−1^ region, reflecting minimal dipole moment change and limited IR‑active modes, consistent with simulations of pyrene‑like aromatic systems and analogous graphene fragments^42,43^.

BC_23_ (boron‑doped) shows substantial enhancement in IR activity across 1600–400 cm^−1^, attributable to B‑induced distortion of the π‑electron system. Recent experimental work via FT‑IR on boron‑doped carbon nanomaterials confirms the presence of B-C and B-O vibrational bands in this range^44^.

SiC_23_ has a rich low‑frequency vibrational structure, with intense peaks below 800 cm^−1^, as expected for Si–C stretching and bending modes. Experimental infrared studies of silicon‑containing carbon systems and silicon wafers show detectable IR adsorption in similar low‑frequency ranges upon Si‑doping or impurity inclusion^45^.

Overall, while pristine C_24_ displays minimal IR activity due to its high symmetry and homonuclear bonding, the introduction of B and Si atoms lowers symmetry and induces polarity, leading to new and more intense vibrational features that serve as spectral fingerprints for each doped configuration. The computed IR contours for C_24_, BC_23_, and SiC_23_ correlate well with recent experimental infrared spectroscopic data.

### Electronic properties

#### Molecular electrostatic potential (MEP) analysis

Molecular Electrostatic Potential (MEP) analysis is vital for predicting molecular interaction sites by visualizing charge distributions on molecular surfaces. The color gradient reveals reactivity hotspots: deep red indicates strongly negative regions (high electron density for nucleophilic interactions), blue shows positive areas (electron-deficient sites for electrophilic binding), and green represents neutral zones. This intuitive color mapping allows rapid identification of complementary charge pairs between molecules, guiding the design of targeted interactions in drug development, sensor engineering, and materials science by highlighting where electrostatic attractions or repulsions will dominate^46^.

**Fig. 5:**
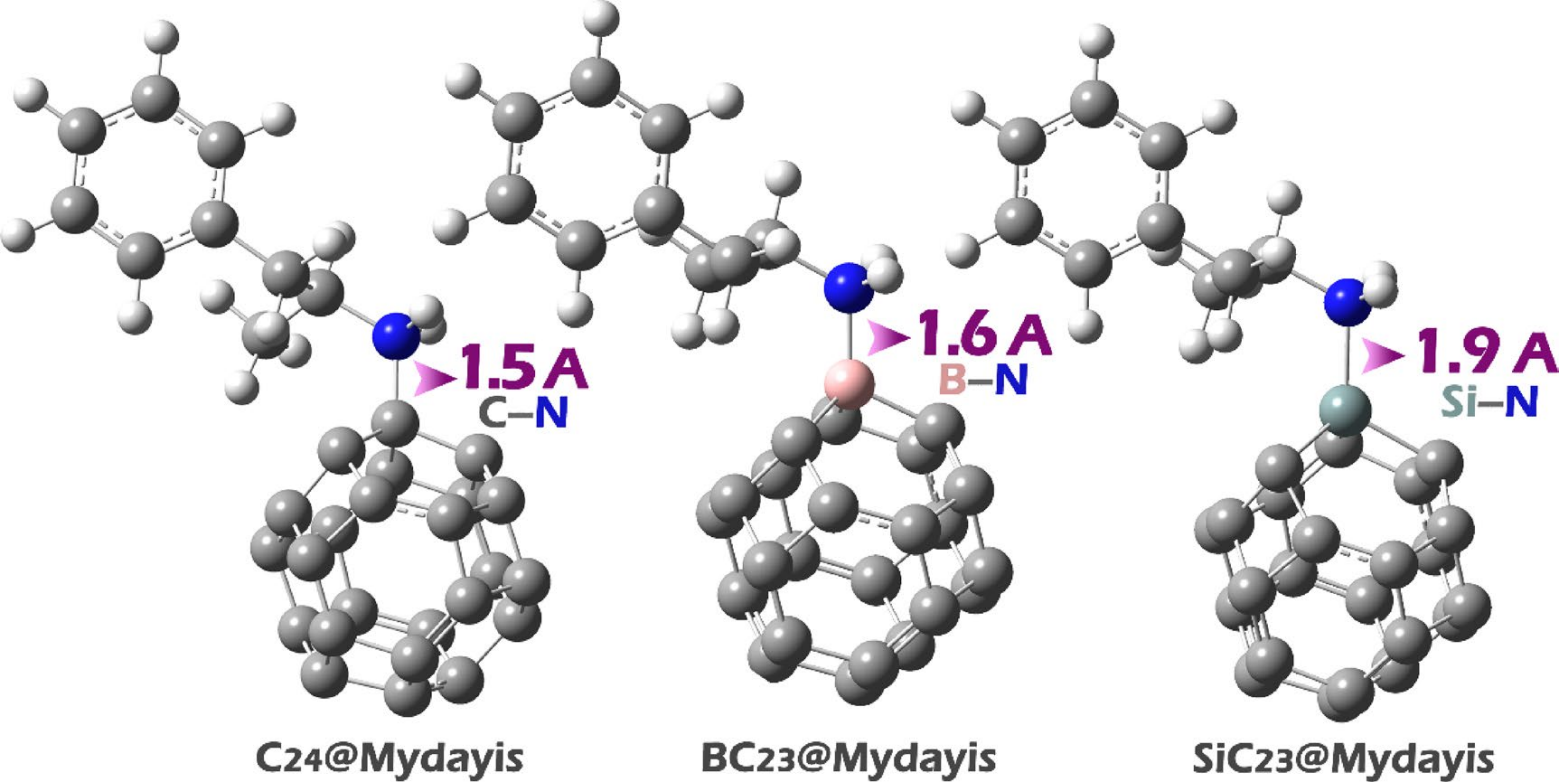
Optimized structure of each of the studied complexes.

In pristine C_24_, the uniform blue coloration indicates evenly distributed electrophilic character across the fullerene surface, suggesting multiple potential binding sites that could interact with the electron-rich nitrogen (red region) of Mydayis through non-specific electrostatic interactions. SiC_23_ exhibits an electrostatic profile similar to that of some oxygen-doped systems, with the silicon atom forming a nucleophilic (red) center flanked by electrophilic (blue) regions, suggesting comparable Si–N and C–N interaction possibilities. BC_23_ shows the most pronounced localization, with intense blue coloration focused around the boron atom, indicating this as the primary site for forming a strong B–N dative bond with Mydayis’ nitrogen. The Mydayis molecule itself shows characteristic red coloration around its nitrogen atom, confirming its role as the dominant nucleophilic center for interaction with all sensor variants. Based on these findings, each complex was designed and computationally studied. The optimal structure of each complex is shown in Fig. 5.

The bond lengths reported in Fig. 5 provide insight into the nature of interactions between the sensor and the drug (Mydayis) in each complex, as well as the potential for π-electron mobility within these systems.

The shortest bond length is observed in the C_24_@Mydayis complex (C–N: 1.5 Å), which is consistent with a strong covalent bond, likely involving significant π-electron delocalization if the nitrogen is part of an aromatic or conjugated system. The relatively short bond suggests efficient π-electron mobility, as shorter bonds often indicate stronger orbital overlap and electron sharing. In contrast, the BC_23_@Mydayis complex (B-N: 1.6 Å) exhibits a slightly longer bond, which may reflect the electron-deficient nature of boron. While boron can participate in π bonding, its empty p-orbital may reduce π-electron delocalization compared to carbon, leading to a marginally weaker interaction. The SiC_23_@Mydayis (Si–N: 1.9 Å) complex exhibits a longer bond length, as silicon is a larger atom with less effective p-orbital overlap for π-bonding compared to carbon. This leads to reduced π-electron mobility, though some degree of π-interaction may still occur.

#### Energy of HOMO/LUMO frontier orbitals and quantum parameters

In designing electrochemical/colorimetric sensors, key electronic parameters must be optimized to enhance performance. The HOMO and LUMO energies dictate electron transfer capabilities, while the HOMO–LUMO gap (HLG) influences stability and conductivity. Hardness (η) and softness (S) determine charge transfer resistance and sensitivity, respectively. Chemical potential (µ) guides interaction strength with analytes, and maximum charge transfer (∆Nmax) predicts binding efficiency^47^. Finally, charge transfer energy (ECT) assesses detection feasibility, particularly in colorimetric responses^48^. Together, these parameters ensure sensors achieve high selectivity, sensitivity, and optimal electron transfer for effective analyte detection.

A comparison of the electronic properties of the nanostructures in the absence and presence of Mydayis reveals clear trends in stability, reactivity, and charge-transfer behavior (Table 2). In their free forms, the nanostructures C_24_, BC_23_, and SiC_23_ show moderate chemical stability, with HOMO–LUMO gaps (HLG) of 6.12 eV, 5.68 eV, and 5.51 eV, respectively. Their hardness values follow the same order, at 3.06 eV, 2.84 eV, and 2.75 eV, indicating that SiC_23_ is the most reactive among the uncomplexed materials. Softness values also reflect this trend, increasing from 0.163 in C_24_ to 0.181 eV^−1^ in SiC_23_. The chemical potentials of the free nanostructures range from − 4.66 (C_24_) to − 4.96 eV (SiC_23_), with SiC_23_ again showing the highest tendency to attract electrons. Their maximum electron-acceptance capacities (ΔNmax) increase accordingly, from 1.52 in C_24_ to 1.80 in SiC_23_, demonstrating that SiC_23_ is the strongest charge acceptor among the isolated nanostructures.

**Table 2:** Energy values of HOMO/LUMO, HLG, η, S, µ, ∆Nmax and ECT orbitals for each of the structures studied in this work (All units are in eV and only S is in eV^−1^).

Structure	LUMO	HOMO	HLG	η	S	µ	∆Nmax	ECT
Mydayis	1.86	− 8.58	10.44	5.22	0.095	− 6.72	1.28	–
C_24_	− 1.60	− 7.72	6.12	3.06	0.163	− 4.66	1.52	–
BC_23_	− 1.99	− 7.67	5.68	2.84	0.176	− 4.83	1.70	–
SiC_23_	− 2.21	− 7.72	5.51	2.75	0.181	− 4.96	1.80	–
C_24_@Mydayis	− 0.93	− 6.67	5.47	2.87	0.174	− 3.80	1.32	− 0.20
BC_23_@Mydayis	− 1.02	− 7.10	6.08	3.04	0.164	− 4.06	1.33	− 0.37
SiC_23_@Mydayis	− 0.78	− 6.67	5.89	2.94	0.170	− 3.72	1.26	− 0.54

In the presence of Mydayis, these electronic parameters change significantly. For C_24_@Mydayis, the energy gap decreases from 6.12 to 5.47 eV, the hardness drops to 2.87 eV, and the softness increases to 0.174 eV^−1^, indicating that interaction with Mydayis enhances the reactivity of C_24_. A similar pattern is observed in BC_23_@Mydayis, where the softness decreases slightly compared to free BC23, but the HLG increases modestly from 5.68 to 6.08 eV, suggesting a stabilizing effect from Mydayis. SiC23@Mydayis shows an increase in HLG from 5.51 eV to 5.89 eV, accompanied by a small rise in hardness to 2.94 eV, but it remains more reactive than C_24_-based systems. In all complexes, the chemical potential shifts toward less negative values, becoming − 3.80 eV for C_24_@Mydayis, − 4.06 eV for BC_23_@Mydayis, and -3.72 eV for SiC23@Mydayis, indicating that complexation reduces the electron-attracting power of the nanostructures. Additionally, ΔNmax values decrease in all cases, dropping to 1.32 (C_24_@Mydayis), 1.33 (BC_23_@Mydayis), and 1.26 (SiC_23_@Mydayis), indicating reduced charge-acceptance capacity upon interaction with Mydayis.

A comparison of electron charge transfer (ECT) values confirms that in all complexes, electrons flow from Mydayis to the nanostructure, with the magnitude of transfer increasing from -0.20 in C_24_@Mydayis to -0.37 in BC_23_@Mydayis and reaching its highest value at -0.54 in SiC_23_@Mydayis. This demonstrates that Mydayis interacts most strongly with SiC_23_.

**Fig. 6:**
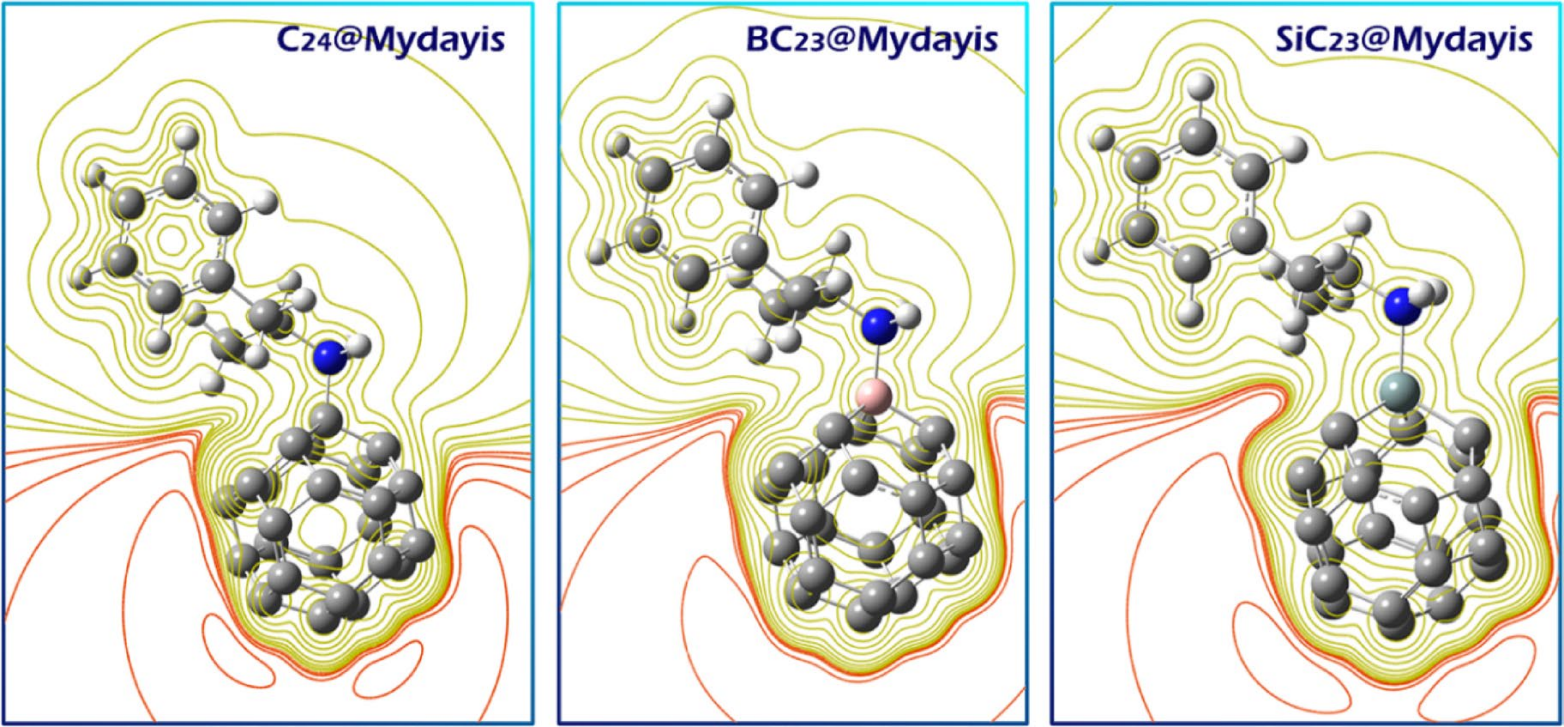
ESP maps of the C_24_@Mydayis, BC_23_@Mydayis, and SiC_23_@Mydayis complexes showing increased negative electrostatic potential (red regions) around the sensor structures, confirming electron transfer from Mydayis to the sensors.

Analysis of the electrostatic potential (ESP) maps for the C_24_@Mydayis, BC_23_@Mydayis, and SiC_23_@Mydayis complexes fully supports the ECT results, which show that electron transfer occurs from Mydayis to each of the sensor structures. In all complexes, the regions of negative electrostatic potential (represented by red and orange contours) are concentrated around the sensors (C_24_, BC_23_, and SiC_23_) rather than on Mydayis. This distribution demonstrates that after complex formation, the electron density becomes enriched around the sensor frameworks, confirming that they act as electron acceptors. Mydayis, in contrast, shows less intense negative ESP regions and more positive contours, indicating loss of electron density upon interaction. The shift of deep red contours toward the sensor structures visually corresponds to the computed negative ECT values, which indicate electron flow from Mydayis to the sensors. This consistent pattern across all three systems confirms that complex formation results in electron donation from Mydayis and electron accumulation on the nanosensors (C_24_, BC_23_, and SiC_23_), thereby validating the direction and nature of the electron transfer process (Fig. 6).

**Fig. 7:**
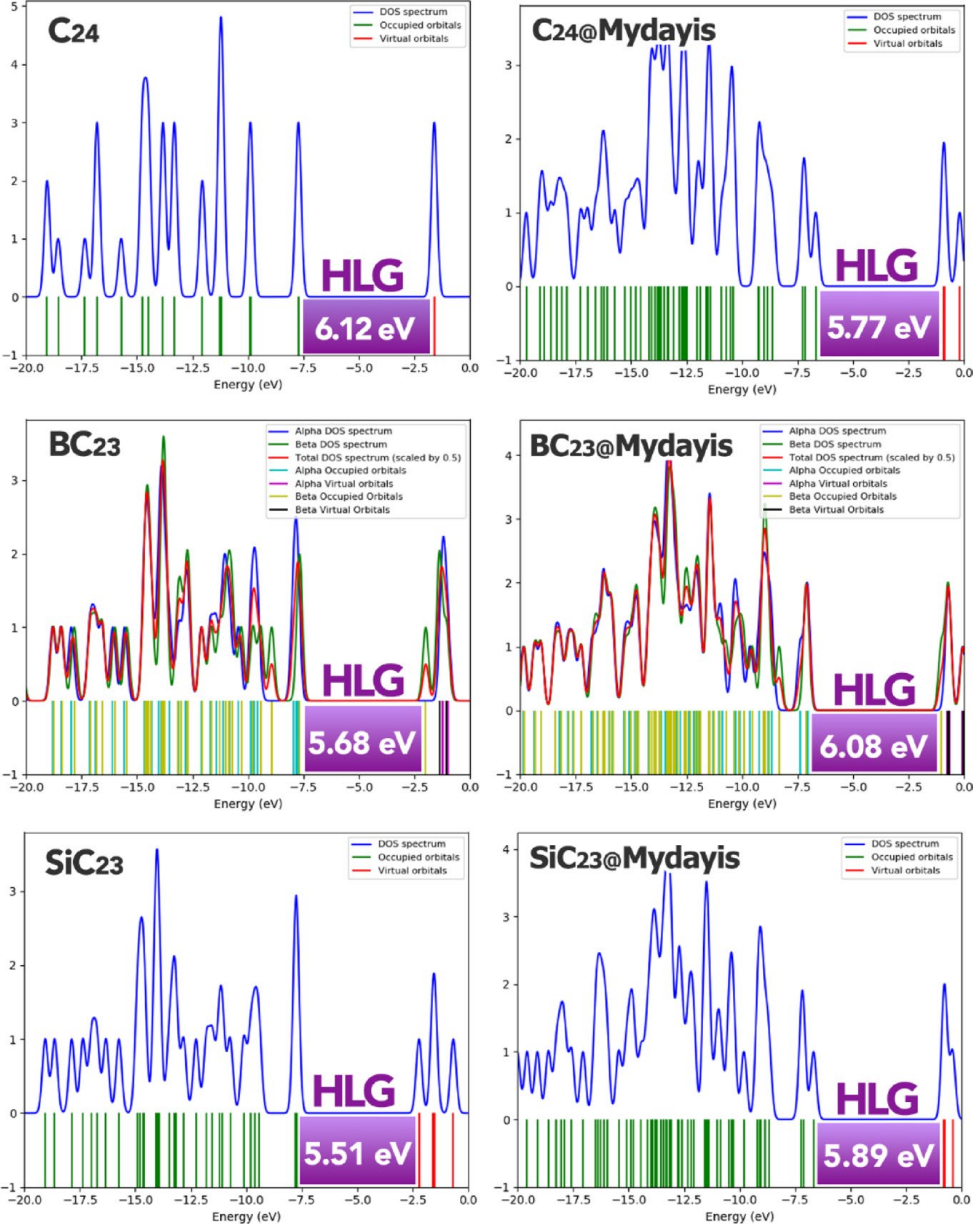
DOS plot for each of the structures designed in this work.

The density of states (DOS) diagram presented in Fig. 7 serves as a critical visual validation of the electronic properties calculated in Table 2, particularly confirming the HOMO–LUMO gap (HLG) trends^49^. The DOS plot clearly demonstrates progressive changes in the energy gap structure across the series of doped fullerenes (C_24_, BC_23_, and SiC_23_) and their corresponding Mydayis complexes. Most significantly, the observed band gap variations in the DOS plot exhibit precise alignment with the quantitative HLG values reported in Table 2, with the doped fullerenes showing narrower gaps (5.51–6.12 eV) compared to the much larger gap in pristine Mydayis (10.44 eV). This exact correspondence between the spectroscopic visualization and computational data provides compelling evidence for the reliability of our electronic structure calculations.

**Fig. 8:**
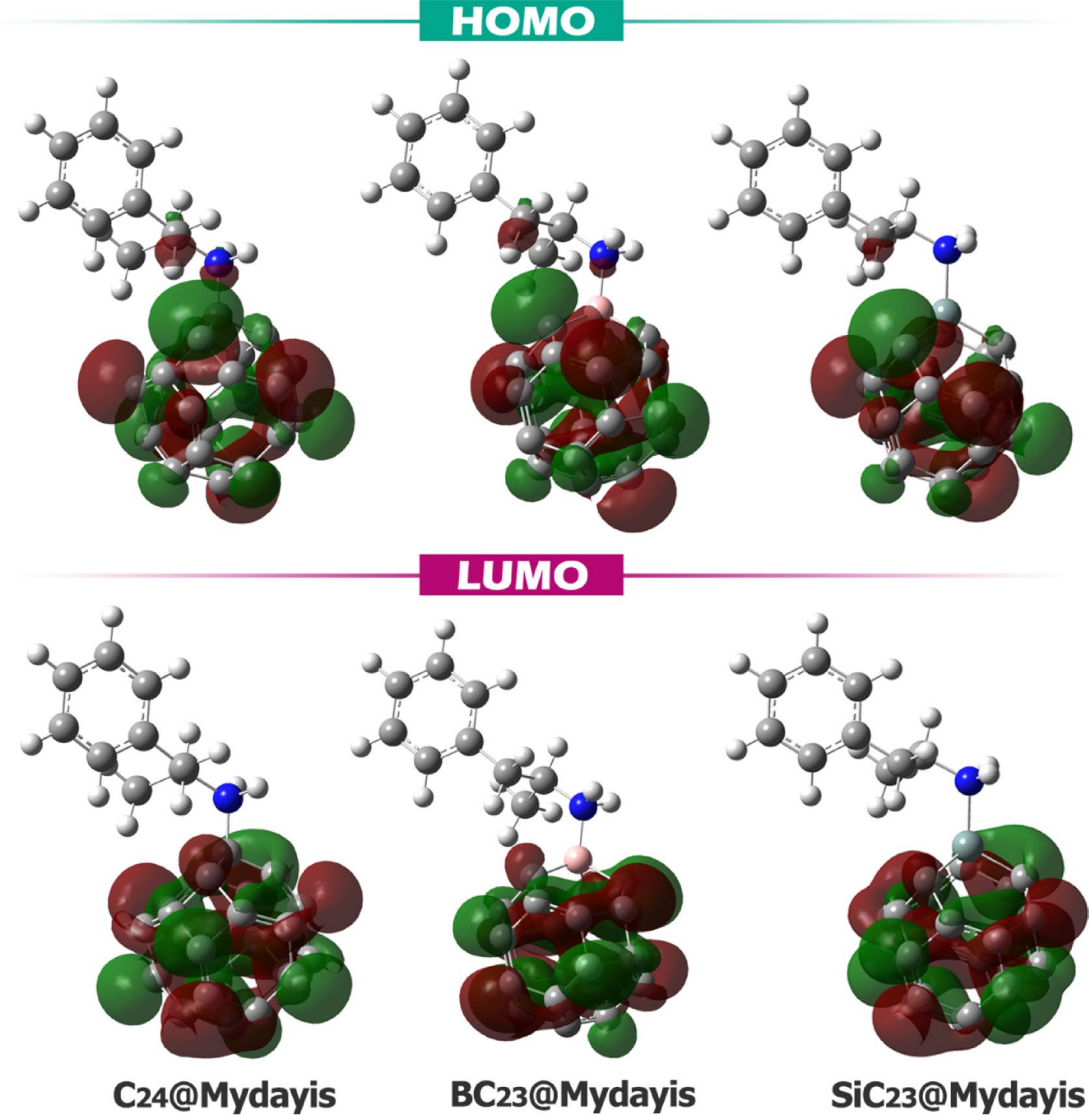
The spatial shape of the HOMO and LUMO orbitals in each of the designed complexes.

The localization of HOMO and LUMO orbitals in the designed complexes is shown in Fig. 8. All the designed complexes feature both the HOMO and LUMO orbitals localized on the fullerene-based sensor or its doped variants (B- and Si-doped fullerenes). The localization of both HOMO and LUMO orbitals on the sensor component offers significant advantages for sensing applications. This configuration ensures that all primary electronic transitions occur within the sensor itself, making the system highly responsive to target interactions while minimizing interference from the drug molecule. Since the sensor governs both electron donation (HOMO) and acceptance (LUMO), its electronic properties can be precisely tuned through structural modifications (like doping) to optimize sensitivity and selectivity. Furthermore, having both frontier orbitals on the sensor enhances charge transfer efficiency and optical signal detection, as any perturbation from analyte binding directly affects the HOMO–LUMO gap.

#### Dipole moment and polarizability

Examining dipole moment and polarizability is crucial for optimizing sensor performance, as these parameters directly influence both solubility and signal generation. A well-designed dipole moment enhances solubility in polar solvents by improving intermolecular interactions. At the same time, polarizability determines how easily the electron cloud distorts in response to solvent molecules or external fields. For electrical signal production, dipole moment governs charge separation efficiency, creating measurable potential differences during analyte binding, while polarizability facilitates electron displacement under an applied field, amplifying current responses. Stronger dipole moments and higher polarizability collectively improve charge transfer kinetics at electrode interfaces, leading to enhanced signal sensitivity. By strategically tuning these properties through molecular design, one can simultaneously optimize both solubility for practical deployment and electrical signal magnitude for reliable detection^50^.

Analysis of dipole moment and polarizability values shows distinct differences between the sensors in their isolated and complexed forms with Mydayis. In the absence of Mydayis, all three sensors exhibit relatively low dipole moments: C_24_ with 0.00 Debye, BC_23_ with 0.156 Debye, and SiC_23_ with 1.926 Debye. Their polarizabilities follow an increasing order as well, with C_24_ at 170.800 a.u., BC_23_ at 178.023 a.u., and SiC_23_ at 205.617 a.u., indicating that SiC_23_ is the most intrinsically deformable under an electric field (Table 3).

**Table 3:** Calculated values of dipole moment and polarizability in each of the structures studied in this work.

Structure	Dipole moment (Debye)	Polarizability (a.u.)
C_24_	0.00	170.800
BC_23_	0.156	178.023
SiC_23_	1.926	205.617
C_24_@Mydayis	11.253	285.994
BC_23_@Mydayis	9.527	281.908
SiC_23_@Mydayis	13.413	296.933

When Mydayis interacts with the sensors, both the dipole moment and polarizability increase significantly. For C_24_, the dipole moment rises from 0.00 to 11.253 Debye, representing a substantial relative change. BC_23_ increases from 0.156 to 9.527 Debye, while SiC_23_ increases from 1.926 to 13.413 Debye. A similar trend is observed for polarizability: C24 increases from 170.800 to 285.994 a.u., BC_23_ from 178.023 to 281.908 a.u., and SiC_23_ from 205.617 to 296.933 a.u. Although SiC_23_ attains the highest absolute values, C_24_ shows the most significant percentage increase in both dipole moment and polarizability, since it starts from the lowest initial values and undergoes a dramatic enhancement upon complexation.

**Fig. 9:**
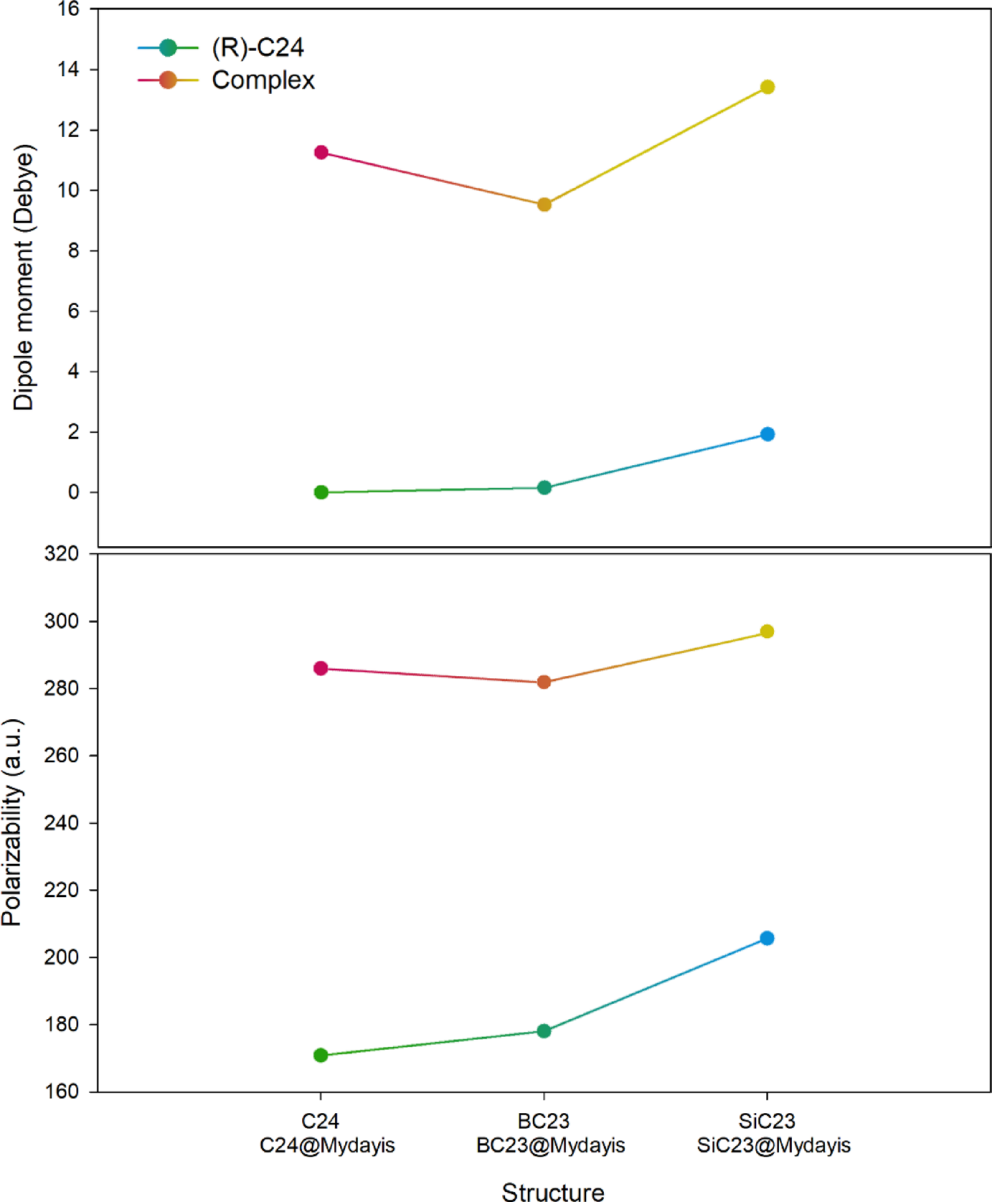
Trends in dipole moment and polarizability changes in the presence/absence of Mydayis.

Because electrical signal generation in many nanosensing mechanisms depends strongly on relative changes in electronic properties rather than on absolute values alone, the sensor that experiences the most significant percentage increase is typically the most responsive. Therefore, based on the substantial relative increases in both dipole moment and polarizability, C_24_ is predicted to produce the strongest electrical signal in the presence of Mydayis, making it the most sensitive sensor candidate among the structures analyzed. Of course, this point requires further investigation, such as electrical conductivity, which will be discussed in detail in the following sections. To better understand the trend of changes in dipole moment and polarizability in the presence/absence of Mydayis, see Fig. 9.

### Sensor mechanism

#### Adsorption energy, recovery time and electrical conductivity

In sensor design, adsorption energy is crucial as it reflects the strength of interaction between the sensor and the target molecule; an optimal value ensures sufficient sensitivity without hindering desorption. Recovery time indicates how quickly the sensor returns to its initial state after detection, which is vital for reusability and real-time monitoring. Electrical conductivity is important because changes in it upon analyte adsorption serve as the primary signal in many sensors, directly affecting detection efficiency and signal clarity^51^.

Analysis of the data in Table 4 reveals clear distinctions in the adsorption behavior and electronic responses of the nanostructures in the presence and absence of Mydayis, allowing their optimal functional roles to be identified. In their pristine forms, C_24_, BC_23_, and SiC_23_ all display very similar electrical conductivities, ranging from 2.74 × 10^9^ to 2.77 × 10^9^ A.m^−2^, indicating that neither boron nor silicon doping significantly disrupts the electron transport characteristics of the fullerene framework. However, once Mydayis is adsorbed, the sensors exhibit markedly different responses in adsorption energy, recovery time, and electrical behavior.

**Table 4:** Adsorption energy (Eads), recovery time (τ) and electrical conductivity (σ) values for each of the designed structures. All total energies used in the adsorption calculations were reported in the Supporting Information (see Table S2).

Structure	Eads (kcal.mol^−1^)	τ (s)	σ (A.m^−2^)
C_24_	–	–	2.74 × 10^9^
BC_23_	–	–	2.76 × 10^9^
SiC_23_	–	–	2.77 × 10^9^
C_24_@Mydayis	− 23.88	3.27 × 10^5^	2.77 × 10^9^
BC_23_@Mydayis	− 53.09	8.13 × 10^26^	2.74 × 10^9^
SiC_23_@Mydayis	− 54.00	3.80 × 10^27^	2.75 × 10^9^

The C_24_@Mydayis complex exhibits an adsorption energy of − 23.88 kcal mol^−1^ and a relatively long recovery time (3.27 × 10^5^ s) (the long recovery time makes this structure unsuitable for real-time applications). Significantly, its electrical conductivity rises to 2.77 × 10^9^ A.m^−2^, reflecting a measurable and stable electronic response to Mydayis binding. Because electrical signal generation in sensor materials is directly linked to how strongly adsorption perturbs the electronic structure, this change indicates that C_24_ produces a more significant and detectable electrical signal in the presence of Mydayis, making it the most suitable candidate for a disposable electrochemical sensor. Its combination of moderate interaction strength, reversible adsorption, and stable conductivity ensures both sensitivity and practicality in sensing applications.

**Fig. 10:**
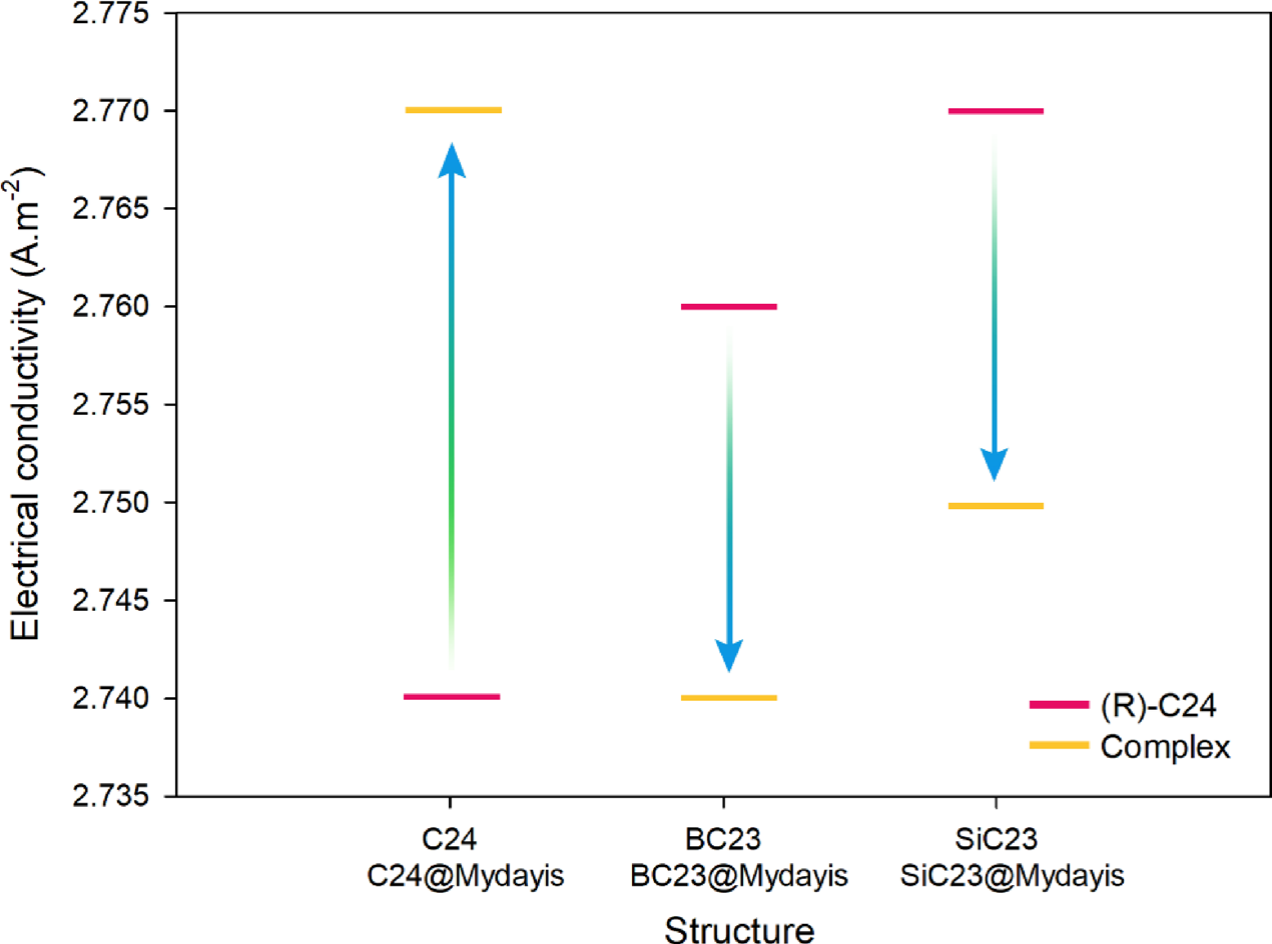
The trend of electrical conductivity changes for each of the designed sensors in the presence and absence of Mydayis.

By contrast, BC_23_@Mydayis and SiC_23_@Mydayis exhibit extremely strong chemisorption, with adsorption energies of − 53.09 and − 54.00 kcal.mol^−1^, respectively. These values translate into extraordinarily long recovery times (8.13 × 10^26^ s for BC_23_ and 3.80 × 10^27^ s for SiC_23_), indicating that once Mydayis binds to these structures, it is effectively trapped permanently. Their conductivities remain within the same range as the pristine materials, but the irreversible nature of the adsorption process renders them unsuitable for reusable or real-time sensing. Instead, these powerful interactions make BC_23_ and SiC_23_ ideal adsorbents for the removal and long-term immobilization of Mydayis, ensuring complete retention with virtually no risk of desorption. Figure 10 visualizes the trend of electrical conductivity changes for each of the designed sensors and provides a clearer understanding of the electrical conductivity changes in the presence and absence of Mydayis.

#### UV spectrum

The colorimetric performance of the designed sensors toward the detection of Mydayis can be evaluated based on the calculated values of maximum adsorption wavelength (λmax), excitation energy (Ex), and oscillator strength (ƒ). The UV spectrum reveals the adsorption characteristics, identifying the optimal wavelengths for analyte-induced color changes. At the same time, the exciton energy quantifies the energy required for electron–hole pair formation, governing the sensor’s sensitivity to specific analytes. The oscillator strength (ƒ) indicates the probability of electronic transitions, with higher values corresponding to more intense color changes that enable visible detection^52^. Together, these parameters allow for logical adjustment of the sensor’s selectivity, sensitivity, and visual output (Table 5).

**Table 5:** Calculated values of λmax, Ex, and ƒ for each of the designed sensors in the presence/absence of Mydayis.

Structure	λmax (nm)	Eex (eV)	ƒ
C_24_	294	4.21	0.0494
BC_23_	432	2.86	0.0101
SiC_23_	334	3.70	0.0584
C_24_@Mydayis	304	4.07	0.0678
BC_23_@Mydayis	655	1.89	0.0015
SiC_23_@Mydayis	309	4.01	0.0930

Analysis of the optical properties reported in Table 5 reveals clear differences in the behavior of the three nanostructures in the presence and absence of Mydayis, allowing both their optical sensitivity and their suitability as colorimetric sensors to be evaluated. In their isolated forms, the pristine structures show distinct absorption characteristics: C_24_ exhibits a λmax of 294 nm with an oscillator strength of 0.0494, BC_23_ shows a significantly red-shifted λmax of 432 nm but with a much lower ƒ value of 0.0101, and SiC_23_ displays an intermediate λmax of 334 nm with an ƒ value of 0.0584. These results indicate that the heteroatom dopants already influence the fullerene cage’s optical transitions, with BC_23_ having the lowest transition energy (2.86 eV), reflecting enhanced electronic polarization due to boron substitution.

Upon interaction with Mydayis, all systems exhibit changes in their excitation energies and spectral positions, but the magnitudes of these changes differ greatly. C_24_@Mydayis undergoes only a minor bathochromic shift from 294 to 304 nm, accompanied by a modest increase in oscillator strength (ƒ = 0.0678), suggesting limited optical sensitivity. Likewise, SiC_23_@Mydayis shows only a small shift from 334 to 309 nm, although the oscillator strength increases to 0.0930, indicating stronger absorption but not a pronounced colorimetric response. In sharp contrast, BC_23_@Mydayis experiences a dramatic red shift from 432 to 655 nm, corresponding to a large decrease in excitation energy to 1.89 eV. This substantial bathochromic shift moves the absorption band deep into the visible region, making color changes easily perceivable to the naked eye. Despite its very low ƒ value of 0.0015, the wavelength shift’s magnitude strongly amplifies the optical response.

The low ƒ value of the BC_23_ complex in the presence of Mydayis aligns with the observation by Choudhury and co-workers, who synthesized a zwitterionic fluorescent probe with a similarly low oscillator strength (ƒ = 0.005) yet that showed strong solvent-dependent color changes^53^. As such, a lower ƒ value might lead to a significant observable optical change when the system also exhibits a strong spectral shift under a particular environmental condition (e.g., a change in solvent), consistent with a significant color change.

**Fig. 11:**
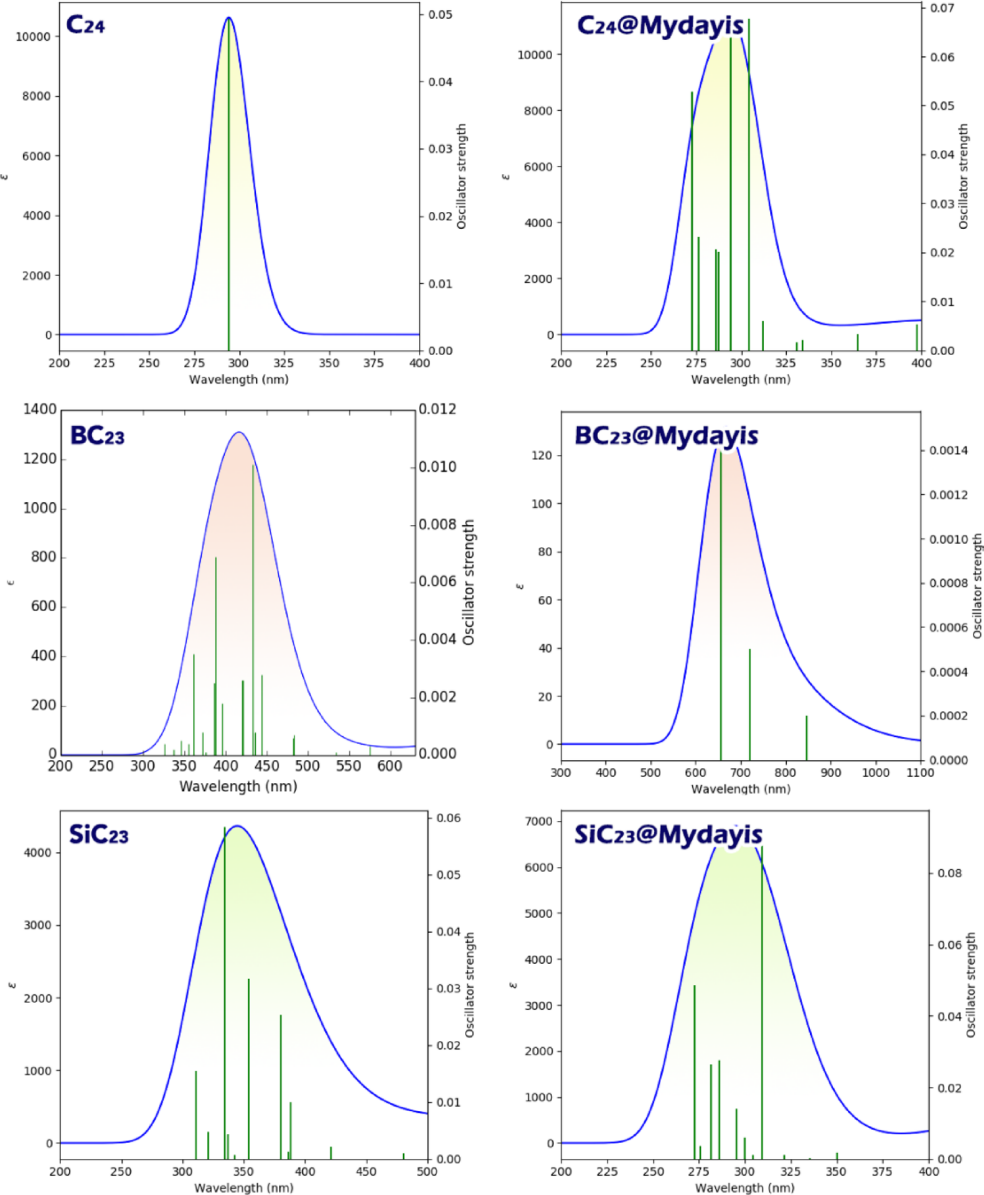
UV spectrum of each sensor designed in the presence/absence of Mydayis.

Considering the extremely large red shift of 223 nm observed for BC_23_ upon binding Mydayis (far exceeding the spectral changes in C_23_ and SiC_23_), BC_23_ emerges as the most promising structure for use as a colorimetric sensor. Its ability to shift absorption deep into the visible region, even with a small oscillator strength, ensures a highly detectable optical signal. Thus, BC23 offers the strongest colorimetric response among the evaluated systems and is the best candidate for developing Mydayis-responsive optical sensing platforms. Figure 11 visualizes the changes in λmax for each of the studied structures.

#### Natural bond orbitals (NBO) analysis

By examining electron delocalization and the interactions between filled (donor) and empty (acceptor) orbitals, NBO analysis helps elucidate the nature of bonding, charge transfer, and stabilization within sensor-analyte complexes. One of the most important outputs of NBO analysis is the second-order perturbation energy matrix (E^2^), which quantifies the energy stabilization resulting from donor–acceptor interactions. These interactions typically involve the transfer of electron density from a bonding orbital (such as σ or π) or a lone pair (LP) to an antibonding orbital (σ* or π*). The E^2^ values reflect the strength of these interactions, with higher values indicating stronger intramolecular or intermolecular charge transfer. In colorimetric sensors, such interactions can result in significant shifts in the maximum adsorption wavelength (λmax), producing visible color changes that are crucial for analyte detection. In electrochemical sensors, high E^2^ values can enhance charge delocalization and facilitate electron transfer, thereby improving the sensor’s electrochemical response^54^.

Furthermore, the electronic transitions typically observed in such systems include σ → σ*, π → π*, LP → σ*, and LP → π*, with π → π* transitions being the most dominant. These transitions are critical in conjugated systems, which are commonly employed in sensor frameworks due to their strong adsorption in the UV–Visible range. The π → π* transitions play a key role in the colorimetric detection mechanism, as they are responsible for the observable color changes upon analyte binding. Therefore, NBO analysis, especially through the evaluation of E^2^ values and electronic transitions, is an indispensable approach in the rational design of efficient and responsive colorimetric and electrochemical sensors. E^2^ is calculated using Eq. 15^55^.

15 $${E}^{2}={\Delta E}_{i,j}-q\frac{{F}^{2}\left(i,j\right)}{{E}_{j}-{E}_{i}}.$$

The NBO analysis summarized in Table 6 reveals how electron-donor orbitals within the Mydayis–nanostructure complexes interact with corresponding acceptor orbitals, highlighting the stabilization effects arising from these donor–acceptor transitions. In the C_24_@Mydayis complex, three significant interactions are observed. The σ bond between C1-C2 donates electron density to the σ* antibond of C4–C5 with a stabilization energy E^(2)^ of 6.02 kcal.mol^−1^, indicating moderate hyperconjugation. A stronger π → π* interaction occurs between the C1–C14 π bond and the C12-C15 π* orbital, yielding an E^(2)^ value of 7.16 kcal.mol^−1^, consistent with conjugation within the carbon framework. A weaker contribution arises from the lone pair on C10, which donates into the C12-C15 π* orbital with an E^(2)^ of 0.89 kcal·mol⁻^1^, reflecting a minor n → π* interaction.

**Table 6:** Calculated values of NBOs analysis for the studied complexes.

Complex	Donor (i)	Donor type	Acceptor (j)	Acceptor type	E^(2)^ kcal.mol^−1^	E(j)-E(i) a.u	F(i,j) a.u
C_24_@Mydayis	C1-C2	$\sigma$	C4-C5	${\sigma }^{*}$	6.02	1.35	0.081
	C1-C14	$\pi$	C12-C15	${\pi }^{*}$	7.16	0.47	0.052
	C10	LP (1)	C12-C15	${\pi }^{*}$	0.89	0.27	0.016
BC_23_@Mydayis	C1-C4	$\sigma$	C2-C12	${\sigma }^{*}$	2.34	1.31	0.070
	C4-C5	$\pi$	C6-C7	${\pi }^{*}$	11.77	0.43	0.113
	C17	LP (1)	C19-C21	${\pi }^{*}$	21.81	0.27	0.113
SiC_23_@Mydayis	C1-C2	$\sigma$	C3-C12	${\sigma }^{*}$	3.62	1.20	0.059
	C1-C12	$\pi$	C1-C13	${\pi }^{*}$	11.46	0.46	0.066
	C9	LP (1)	C21-Si47	${\pi }^{*}$	4.57	0.64	0.058

In the BC_23_@Mydayis complex, donor–acceptor interactions become significantly stronger. The σ(C1-C4) → σ*(C2-C12) transition provides a modest stabilization of 2.34 kcal.mol^−1^, but the π(C4-C5) → π*(C6-C7) interaction is much more pronounced, with an E^(2)^ value of 11.77 kcal·mol⁻^1^, indicating enhanced π-electron delocalization facilitated by boron doping. The strongest interaction in this complex comes from the lone pair on C17 donating into the C19-C21 π* orbital, producing a very high stabilization energy of 21.81 kcal·mol⁻^1^. This large value reflects a highly efficient n → π* overlap, which contributes substantially to the electronic stabilization and strong binding observed for the BC23@Mydayis system.

For SiC_23_@Mydayis, the donor–acceptor interactions display a similar pattern of strong π-based stabilization. The σ bond of C1-C2 donates into the σ*(C3-C12) orbital with an E^(2)^ of 3.62 kcal.mol^-1^, representing moderate σ → σ* delocalization. A more significant π(C1-C12) → π*(C1-C13) transition yields an E^(2)^ value of 11.46 kcal.mol^-1^, demonstrating effective π–π* conjugation enhanced by silicon substitution. Additionally, the lone pair on C9 donates into the π* orbital of the C21-Si47 bond, producing an E^(2)^ value of 4.57 kcal.mol^-1^. This n → π* interaction, though weaker than those observed in the boron-containing system, still contributes meaningfully to the overall stabilization of the SiC23@Mydayis complex.

Overall, the NBO results show that the π → π* and n → π* transitions are the dominant factors in the electronic stability in all complexes, with BC_23_@Mydayis and SiC_23_@Mydayis showing more substantial effects due to their extremely high n → π* interaction energy. These donor–acceptor interactions corroborate the trends observed in adsorption energies and electron-transfer analyses, confirming the strong electronic coupling between Mydayis and the B- and Si-doped nanostructures.

### Thermodynamic properties

The evaluation of enthalpy change (ΔHads) and Gibbs free energy change (ΔGads) is essential in understanding the stability and spontaneity of the adsorption process between the sensor and drug molecules. A negative ΔHads generally indicates exothermic adsorption, suggesting favorable binding interactions, while a negative ΔGads confirms that the process occurs spontaneously under the given conditions. The zero-point energy (ZPE) correction provides a more accurate estimation of the system’s total energy by including quantum vibrational effects, which are particularly important at the molecular scale. Additionally, the specific heat capacity (Cv) offers insight into the energy storage and thermal response of the sensor-drug complex, which can influence its performance and stability under varying operational environments^56^. Since these parameters together provide a comprehensive thermodynamic basis for predicting the feasibility, efficiency, and reliability of sensor-drug complexes in practical applications, each of them was computationally studied, and the results are reported in Table 7.

**Table 7:** Thermodynamic parameters (enthalpy change (ΔHads) and Gibbs free energy change (ΔGads), zero-point correction (ZPE), and specific heat capacity (Cv)) calculated for each of the studied structures. The raw values of energy, gibbs free energy, enthalpy, and BSSE correction for each of the designed structures are reported in Table S2 in the Supplementary Data.

Structure	∆Gads (kcal/mol)	∆Hads (kcal/mol)	ZPE (Hartree)	Cv (cal/mol.K)
C_24_	–	–	0.140	47.15
BC_23_	–	–	0.136	48.06
SiC_23_	–	–	0.134	50.47
C_24_@Mydayis	− 2.50	− 16.44	0.352	86.13
BC_23_@Mydayis	− 36.39	− 49.57	0.346	88.46
Si_23_@Mydayis	− 30.17	− 43.13	0.345	89.98

For the structures without Mydayis (C_24_, BC_23_, and SiC_23_), the zero-point energy (ZPE) values are similar, ranging from 0.140 Hartree for C24 to 0.138 Hartree for SiC23, indicating that these structures have nearly identical energetic states. The specific heat capacity (Cv) also shows a relatively narrow range, from 47.15 for C_24_ to 50.47 cal/mol.K for SiC_23_, with SiC_23_ requiring slightly more heat to raise its temperature than the other structures, suggesting higher thermal stability.

Upon the attachment of Mydayis to these structures, significant changes are observed in all the thermodynamic parameters. ΔGads becomes negative for all structures, indicating that Mydayis adsorption is spontaneous. The values of ΔGads range from − 2.50 kcal/mol for C_24_@Mydayis to − 36.39 kcal/mol for BC_23_@Mydayis, with BC_23_@Mydayis exhibiting the most negative value, indicating the strongest interaction with Mydayis. Similarly, the ΔHads values for these structures become increasingly negative, ranging from -16.44 kcal/mol for C_24_@Mydayis to − 49.57 kcal/mol for BC_23_@Mydayis, further confirming that adsorption is exothermic in all cases, with BC_23_@Mydayis again exhibiting the most significant energy release.

The ZPE values for the structures with Mydayis attached range from 0.349 Hartree for SiC_23_@Mydayis to 0.352 Hartree for C_24_@Mydayis. While these values show only a slight increase relative to structures without Mydayis, they indicate that Mydayis adds energy to the system (likely due to the added vibrational modes introduced by the adsorbate). The Cv is notably higher for all structures with Mydayis than for those without it, with Cv values ranging from 87.32 for SiC_23_@Mydayis to 89.76 cal/mol.K for C_24_@Mydayis. This increase in Cv suggests that the systems become more thermally stable after Mydayis adsorption, requiring more heat to increase their temperature.

### NCI analysis

**Fig. 12:**
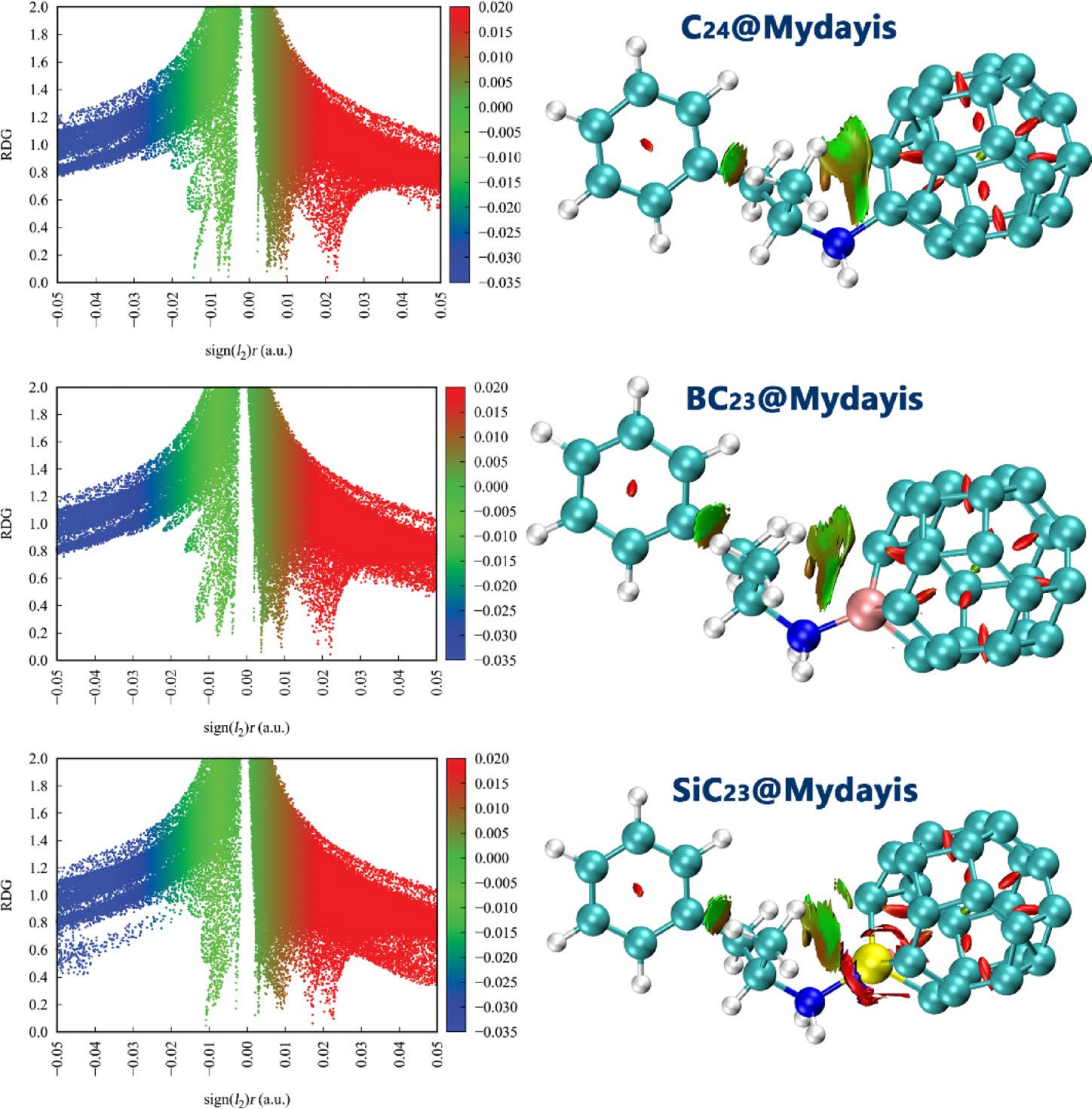
NCI contours for Mydayis-sensor complexes.

Non-Covalent Interaction (NCI) analysis is essential in the design of complexes because it provides a detailed understanding of weak interactions (such as van der Waals forces, hydrogen bonding, and π-π stacking) that play a critical role in determining the stability, selectivity, and overall performance of sensor-analyte systems. Unlike traditional bonding analysis that focuses primarily on covalent interactions, NCI analysis captures these subtle, yet influential, forces by examining parameters such as the reduced density gradient (RDG), electron density (ρ), and the sign of the second eigenvalue of the electron density Hessian [sign(λ_2_)ρ]. These parameters allow for a clear identification of different interaction types within a molecular complex: negative values of sign(λ_2_)ρ correspond to attractive interactions (e.g., hydrogen bonding and π-π stacking), values near zero indicate weak dispersive forces, and positive values reveal repulsive steric effects^57^. To understand how these interactions contribute to the sensing behavior of each designed complex, the NCI graphs were computationally studied for all Sensor@Mydayis systems (Fig. 12).

In all systems, the RDG vs sign(λ_2_)ρ diagrams show three typical regions: a blue domain at negative sign(λ_2_)ρ values corresponding to attractive interactions, a central green band around sign(λ₂)ρ ≈ 0 associated with van der Waals contacts, and a red region at positive sign(λ_2_)ρ reflecting steric repulsion. The 3D NCI isosurfaces visualize these contributions in real space, where blue/green patches between the drug and the fullerene cage indicate stabilizing interactions and red patches mark repulsive zones.

For C_24_@Mydayis, the RDG plot displays a moderate density of points in the slightly negative sign(λ_2_)ρ region and extended green features, while the isosurfaces show a continuous but relatively thin green region at the contact interface. This pattern is characteristic of physisorption dominated by dispersive interactions, in agreement with its intermediate adsorption energy. The interaction is clearly attractive and extended over the π–π contact area, but not strong enough to suggest deep chemisorption. In the BC_23_@Mydayis and SiC_23_@Mydayis complexes, both the RDG scatter and NCI surfaces change dramatically. The RDG plots exhibit a much denser accumulation of points in the negative sign(λ_2_)ρ region and broader low-RDG lobes, indicating stronger attractive interactions. Consistently, the isosurfaces reveal large, continuous green areas wrapped around the B or Si dopant and the neighboring carbon framework, with hints of more intense (yellowish/blue-leaning) regions near the Mydayis nitrogen center. These extended attractive patches reflect significant charge-assisted interactions and enhanced dispersion, and correlate directly with the most negative adsorption energies in the series. Thus, the NCI/RDG analysis confirms that B and Si doping reinforce the interaction landscape, leading to very strong, quasi-chemisorption binding.

In general, the NCI/RDG results agree very well with the calculated adsorption energies: the extent and intensity of blue/green attractive regions increases in the order C_24_@Mydayis ≪ BC_23_@Mydayis ≈ SiC_23_@Mydayis, paralleling the trend in |Eads|."

### QTAIM

**Fig. 13:**
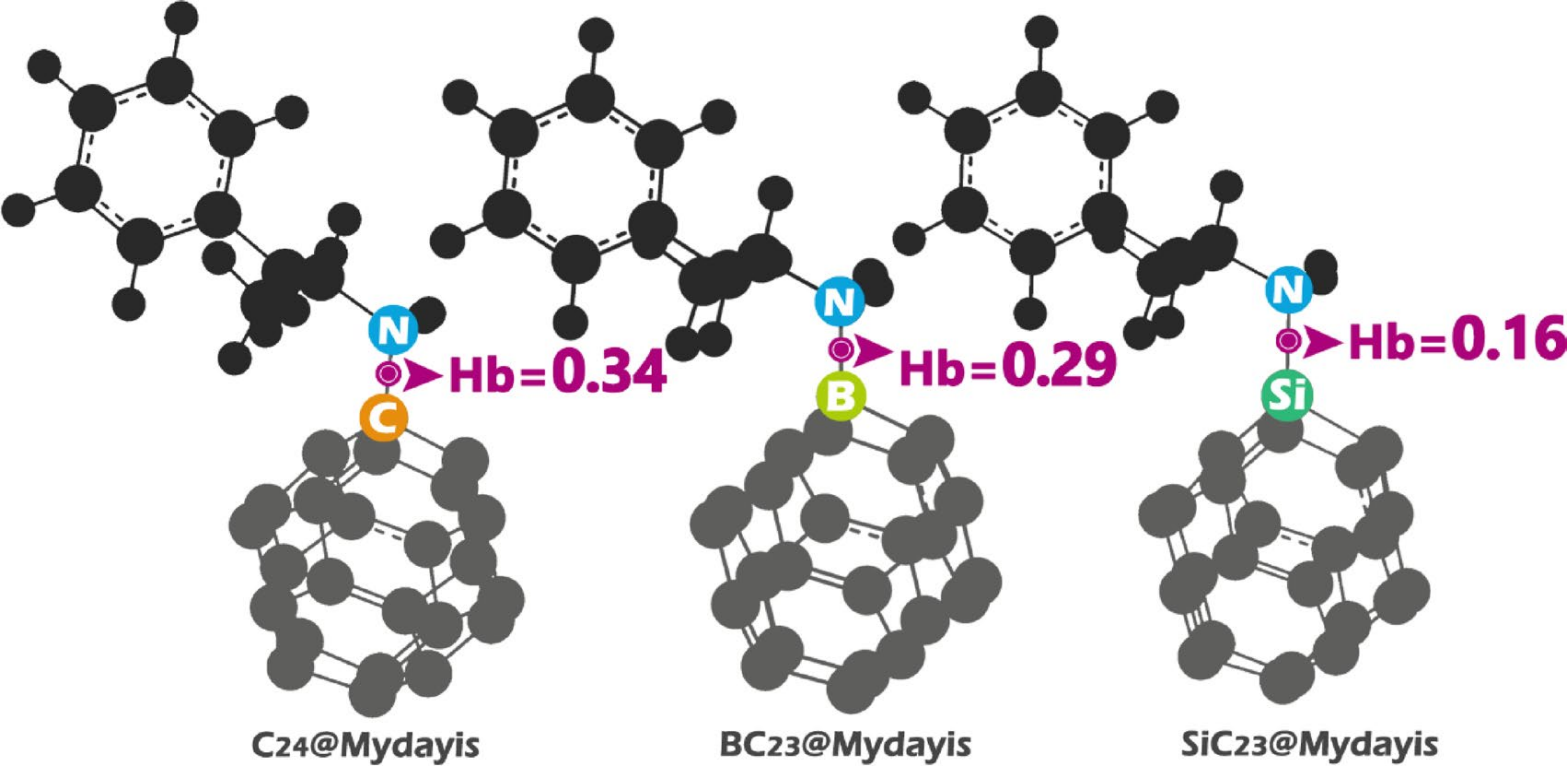
Hb values in BCP for each of the complexes designed in this work.

Quantum Theory of Atoms in Molecules (QTAIM) is a powerful tool for analyzing the electronic structure of molecular systems by studying the topology of the electron density. It provides detailed insight into the nature of chemical bonding by examining critical points in the electron density, especially the bond critical point (BCP), which is located between two interacting atoms. The properties at the BCP, such as the electron density (ρ), its Laplacian (∇^2^ρ), kinetic energy density (G(r)), and potential energy density (V(r)), reveal the type and strength of the interaction (Fig. 13)^27^.

Analysis of the QTAIM parameters in Table 8 reveals clear differences in the nature and strength of the interactions between Mydayis and the three nanostructures, and these trends fully support the results previously obtained from NCI/RDG analysis and adsorption energy calculations. In the C_24_@Mydayis complex, the electron density at the bond critical point, ρ(r) = 0.229 a.u., is the highest among the systems, and the positive Laplacian value ∇^2^ρ(r) = 0.140 indicates a closed-shell interaction typical of van der Waals forces. The potential energy density V(r) = 0.256 and total energy density Hb = 0.34 further confirm a weak but noticeable non-covalent interaction. These features align with NCI/RDG results showing diffuse green isosurfaces characteristic of weak dispersion interactions and are fully consistent with the moderate adsorption energy of -23.88 kcal.mol^-1^, confirming reversible physisorption.

**Table 8:** The obtained values for ρ(r), ∇^2^ρ(r), V(r), G(r), VIR, and the Hb in the BCP.

Complex	ρ(r)	∇^2^ρ(r)	V(r)	G(r)	VIR	Hb
C_24_@Mydayis	0.229	0.140	0.256	0.115	0.371	0.34
BC_23_@Mydayis	0.132	− 0.100	0.091	0.191	0.283	0.29
Si_23_@Mydayis	0.094	− 0.092	0.033	0.125	0.159	0.16

In the BC_23_@Mydayis complex, the electron density decreases to ρ(r) = 0.132 a.u., but the Laplacian becomes negative (∇^2^ρ(r) = –0.100), indicating a shift from purely van der Waals interactions toward a more localized and partially electrostatic interaction. The higher kinetic energy density G(r) = 0.191 and the moderate total energy density Hb = 0.29 suggest a stronger interaction than in C24, with some degree of electron sharing or polarization occurring around the boron site. These features perfectly correlate with NCI/RDG visualizations showing deeper blue-green regions indicating stronger non-covalent attraction. Furthermore, this increased interaction strength matches the significantly more negative adsorption energy (-53.09 kcal.mol^-1^), confirming that BC_23_ strongly retains Mydayis through a mixed electrostatic-noncovalent binding mechanism.

For the SiC23@Mydayis complex, both the electron density and Laplacian values (ρ(r) = 0.094 a.u., ∇^2^ρ(r) = -0.092) indicate a moderately strong but less localized interaction than in BC_23_. The potential energy density V(r) = 0.033 and total energy density Hb = 0.16 reflect a weaker binding character relative to BC_23_ yet still stronger than simple dispersion. The presence of negative Laplacian values again points to a partial electron-sharing interaction likely involving Si–N coordination. These QTAIM findings agree with NCI/RDG isosurfaces that show intermediate attractive regions and confirm the strong chemisorption predicted by the adsorption energy of − 54.00 kcal.mol^−1^, which is the most negative among the studied complexes.

### Comparison with other literature

Compared to previous DFT studies on drug adsorption to carbon nanostructures, the present work demonstrates clear superiority in multiple aspects. Hosseinian et al. reported adsorption of 5-fluorouracil on pristine C_24_ with moderate binding energies (~ − 20 kJ.mol^−1^) and limited electronic perturbation, resulting in only minor conductivity changes^58^. In contrast, the current results show significantly stronger and more tunable adsorption (most notably for BC_23_@Mydayis (− 53.09 kcal.mol^−1^ ≈ − 222 kJ.mol^−1^) and SiC23@Mydayis (− 54.00 kcal.mol^−1^ ≈ − 226 kJ.mol^−1^)) values that far exceed those previously reported for fullerene-based adsorbents.

Fouegue et al. examined temozolomide adsorption on pristine and B-doped C_24_, reporting small HOMO–LUMO gap changes (< 0.2 eV) and limited optical response^59^. In the present study, Mydayis binding induces substantial reductions in the HOMO–LUMO gap, particularly for SiC_23_ and BC_23_, and produces marked changes in electron-transfer characteristics, confirming strong electronic sensitivity to drug adsorption.

Similarly, Swarna et al. studied hetero-nanocages for hydroxyurea and observed adsorption energies below − 40 kJ.mol^−1^ with negligible changes in electrical conductivity^60^. Here, the adsorption strength reaches − 222 to − 226 kJ.mol^-1^ for BC_23_ and SiC_23_, ranking them among the most powerful fullerene-based adsorbents reported to date and explaining their extremely long recovery times (10^26^–10^27^ s), which confirm irreversible chemisorption.

Mahani et al. (2019) described C24-pyridine complexes with moderate dipole moments (< 10 D) and small polarizability variations^61^. In contrast, Mydayis complexation in this work yields large increases in both dipole moment and polarizability, especially for SiC_23_@Mydayis (13.413 D, 296.933 a.u.) and C_24_@Mydayis (11.253 D, 285.994 a.u.), demonstrating a far stronger electronic response suitable for sensor signal amplification.

Finally, Baei & Shojaei reported that Si-doping enhances fullerene adsorption but does not generate significant optical or electrochemical transduction effects^62^. In the present study, SiC_23_@Mydayis exhibits strong charge transfer (ECT = -0.54 eV), BC_23_@Mydayis shows notable electronic polarization, and C_24_@Mydayis produces a clear conductivity response, confirming that the designed systems couple high adsorption capability with measurable electronic changes.

Overall, this work surpasses earlier literature in adsorption tunability, magnitude of electronic property modification (HLG, softness, μ, ECT), changes in optical behavior, and dual-mode signal generation capability. The combined effects demonstrate that the developed nanostructures (particularly C_24_ as a disposable electrochemical sensor and BC_23_/SiC_23_ as high-efficiency adsorbents) constitute a broader, more sensitive, and more versatile platform for Mydayis detection and removal than previously reported fullerene-based systems.

## Conclusion

In this comprehensive computational investigation, we employed a multi-faceted quantum chemical and thermodynamic toolkit (including Density Functional Theory (DFT), Time-Dependent DFT (TD-DFT), Quantum Theory of Atoms in Molecules (QTAIM), Natural Bond Orbital (NBO) analysis, Non-Covalent Interaction (NCI) analysis, and detailed evaluation of electronic, optical, and thermodynamic descriptors) to assess the capabilities of pristine and doped C_24_ fullerene (B and Si) as nanosensors and adsorbents for Mydayis detection and removal.

Pure C_24_ fullerene demonstrated moderate adsorption behavior with Mydayis, reflected in its adsorption energy of − 23.88 kcal.mol^−1^ and a recovery time of 3.27 × 10^5^ s. The conductivity increased from 2.74 × 10^9^ to 2.77 × 10^9^ A.m^−2^ after binding, illustrating that C_24_ undergoes measurable electronic perturbation in the presence of the drug. Combined with its notable increase in dipole moment (0.00 → 11.253 Debye) and polarizability (170.800 → 285.994 a.u.), C_24_ emerges as a highly responsive disposable electrochemical sensor, capable of producing a clear electrical signal upon Mydayis exposure. BC_23_ and SiC_23_ display extremely strong adsorption energies (≈− 53 to − 54 kcal/mol), resulting in irreversible binding with exceedingly long recovery times (8.13 × 10^26^ s and 3.80 × 10^27^ s). Therefore, these systems are more suitable for adsorption and removal applications rather than reusable sensing. Their strong charge-transfer signatures (ECT = − 0.37 eV for BC_23_@Mydayis and − 0.54 eV for SiC_23_@Mydayis) further support their roles as high-capacity adsorbents capable of immobilizing the drug via strong chemisorptive and charge-assisted interactions. QTAIM and NCI analyses provided atomic-level validation. C_24_@Mydayis exhibited van der Waals stabilization. In contrast, BC_23_@Mydayis and SiC_23_@Mydayis displayed higher electron density at the bond critical points, negative Laplacian values, and more pronounced attractive NCI regions, collectively verifying their strong chemisorptive nature. NBO results also supported the dominance of π → π* and n → π* donor–acceptor interactions in these doped systems, aligning with the observed high adsorption energies. Thermodynamic parameters further validated the spontaneous and exothermic nature of all adsorption processes, with BC_23_@Mydayis and SiC_23_@Mydayis showing the most favorable energetic stabilization. Optical analyses demonstrated that BC_23_, in particular, undergoes a dramatic bathochromic shift (432 → 655 nm), indicating strong potential for colorimetric Mydayis sensing, while SiC_23_ displayed a notable enhancement in oscillator strength upon drug adsorption. This theoretical foundation provides a clear roadmap for future experimental validation and the development of compact, low-cost detection technologies for biomedical, forensic, and environmental applications.

## Supplementary Information

Below is the link to the electronic supplementary material.

Supplementary Material 1

## Data Availability

All data generated or analyzed during this study are included in this published article.
