# Supplementary material for: Quantum and thermodynamic evaluation of C24 fullerene-based nanosensors for detection of mydayis in biomedical and drug detection applications
Source: Sci Rep. 2026 Jan 6;16:4481. doi: 10.1038/s41598-025-34744-3 (PMC12865197; doi:10.1038/s41598-025-34744-3)
Supplement: Supplementary file 1 — Supplementary Material 1 [file 41598_2025_34744_MOESM1_ESM.docx]

The X, Y, and Z coordinates for each of the structures designed in this work are provided in Table S1. Including these coordinates ensures full reproducibility of the models and allows all reported values in this study to be independently verified and repeated.

| Table S1. X, Y, Z coordinates for each of the structures designed in this work. |
| --- |
| BC23 |
| %nprocshared=4  %chk=C:\Users\Administrator\Desktop\BC23.chk  # opt wb97xd/6-31g(d) scrf=(cpcm,solvent=water) geom=connectivity out=wfn  Title Card Required  0 2  C 1.70705291 -0.63719363 4.97338428  C 2.03225040 -2.11790363 4.94766452  C 3.53789760 -3.25449729 3.24951651  C 2.91039080 -0.50744179 5.88604965  C 3.99920298 0.56579757 5.63218743  C 5.28594065 -0.05389130 5.11541380  C 5.65591293 -1.51772580 5.20067534  C 4.42160103 -2.43451274 5.51638583  C 3.92835463 1.20578606 4.22226591  C 2.73882271 1.22741843 3.27368454  C 3.01901717 0.36485611 2.02583167  C 1.77967644 -0.52636807 2.24974792  C 2.10716340 -2.90085768 3.61136476  C 1.78174941 0.09850907 3.64096458  C 2.18259636 -1.99066920 2.35727553  C 3.46943861 -2.64570164 1.83459418  C 4.56357360 -1.58514434 1.64827597  C 4.38364603 -0.06065803 1.47677837  C 5.69412719 -2.14139880 3.81643087  C 5.44851759 0.03291907 2.59162480  C 5.85397770 -1.44196320 2.46744370  C 5.38641715 0.86363342 3.88177596  C 4.71590160 -3.23771507 4.19765257  B 2.98790333 -2.14624360 6.37007600  1 2 1.0 4 1.0 14 1.0  2 13 1.0 24 1.0  3 13 1.0 16 1.0 23 1.0  4 5 1.0 24 1.0  5 6 1.0 9 1.0  6 7 1.0 22 1.0  7 8 1.0 19 1.0  8 23 1.0 24 1.0  9 10 1.0 22 1.0  10 11 1.0 14 1.0  11 12 1.0 18 1.0  12 14 1.0 15 1.0  13 15 1.0  14  15 16 1.0  16 17 1.0  17 18 1.0 21 1.0  18 20 1.0  19 21 1.0 23 1.0  20 21 1.0 22 1.0  21  22  23  24  C:\Users\Administrator\Desktop\BC23.wfn |
| BC23@Mydayis |
| %nprocshared=4  %chk=C:\Users\SanCo\Desktop\BC23-Mydayis.chk  # opt wb97xd/6-31g(d) scrf=(cpcm,solvent=water) pop=nbo geom=connectivity  out=wfn  Title Card Required  0 2  C 2.20401500 -0.05349700 -2.15480300  C 3.61604600 0.34167700 -1.94589100  C 4.46577600 1.46298400 0.08220700  C 2.47761800 -1.42159800 -1.52270400  C 1.65403100 -1.96200300 -0.54243600  C 2.16727900 -1.81192100 0.84520500  C 3.48585000 -1.44684900 1.09883600  C 4.39427300 -1.03809500 0.00722300  C 0.16877200 0.53862200 -0.86682400  C 0.61333200 1.67679500 -0.06345800  C 1.59783600 2.08285500 -1.11529300  C 3.93306900 1.50657800 -1.29326800  C 1.18916000 0.85333600 -1.86449200  C 2.87172300 2.47190800 -0.88718900  C 3.35686000 2.33369500 0.51664500  C 2.51232000 1.92377800 1.51768200  C 1.13029100 1.48605400 1.21455900  C 3.81281700 -0.20530100 1.83038900  C 1.36417800 0.17497700 1.97026000  C 2.74207800 0.60613700 2.13276100  C 1.02779700 -1.05820900 1.42602000  C 4.64735700 0.23478000 0.70104700  C 3.81513600 -0.93268100 -1.23731400  C -6.10122700 2.09801400 0.68486300  C -4.87665400 1.48762300 0.42227700  C -4.80629700 0.33713300 -0.36672100  C -5.99107900 -0.19164000 -0.88764800  C -7.21688600 0.41451800 -0.62769500  C -7.27503700 1.56292600 0.16018700  H -6.13659800 2.99445100 1.29708200  H -3.96296800 1.91043200 0.83292400  H -5.95088800 -1.08371900 -1.50872800  H -8.12669600 -0.00722400 -1.04469100  H -8.22999000 2.03966700 0.35991700  C -3.48326500 -0.33780000 -0.63661300  H -3.42639700 -0.62510600 -1.69419100  H -2.66115100 0.36238900 -0.45076500  C -3.27081200 -1.58865800 0.22674200  H -4.12058100 -2.26543000 0.07386400  C -3.15234800 -1.27193400 1.71056700  H -2.33735900 -0.56332300 1.89560700  H -4.08182000 -0.82526600 2.07298100  H -2.96266800 -2.17997300 2.29204600  H -1.93555800 -3.15144800 0.30541500  H -2.17852100 -2.60153300 -1.19604200  N -2.03983900 -2.29175600 -0.23366600  B -0.26846600 -1.32022800 -0.16034900  1 2 1.0 4 1.0 13 1.5  2 12 2.0 23 1.0  3 12 1.0 15 1.0 22 1.5  4 5 1.5 23 1.0  5 6 1.0 47 1.0  6 7 1.5 21 1.0  7 8 1.0 18 1.0  8 22 1.0 23 2.0  9 10 1.0 13 1.0 47 1.0  10 11 1.0 17 1.5  11 13 1.0 14 2.0  12 14 1.0  13  14 15 1.0  15 16 2.0  16 17 1.0 20 1.0  17 19 1.0  18 20 2.0 22 1.0  19 20 1.0 21 1.5  20  21 47 1.0  22  23  24 25 1.5 29 1.5 30 1.0  25 26 1.5 31 1.0  26 27 1.5 35 1.0  27 28 1.5 32 1.0  28 29 1.5 33 1.0  29 34 1.0  30  31  32  33  34  35 36 1.0 37 1.0 38 1.0  36  37  38 39 1.0 40 1.0 46 1.0  39  40 41 1.0 42 1.0 43 1.0  41  42  43  44 46 1.0  45 46 1.0  46 47 1.0  47  C:\Users\SanCo\Desktop\BC23-Mydayis.wfn |
| C24 |
| %nprocshared=4  %chk=C:\Users\SanCo\Desktop\C24.chk  # opt wb97xd/6-31g(d) scrf=(cpcm,solvent=water) geom=connectivity out=wfn  Title Card Required  0 1  C -0.68755836 -1.45841751 -2.20591594  C 0.70502764 -1.08334551 -1.85159094  C 1.56131164 0.06383449 0.10641206  C -0.46371836 -2.69408451 -1.38963594  C -1.27030936 -3.11186051 -0.37205894  C -0.69529536 -3.02884551 0.96818306  C 0.64239664 -2.80277351 1.24872106  C 1.44883064 -2.36103551 0.08971806  C -2.51482436 -2.28125851 0.06752706  C -2.61918536 -0.93870151 -0.62970994  C -2.15806436 0.23659249 0.08124206  C -1.33529136 0.71265949 -1.06484194  C 1.08707264 0.11798849 -1.29151094  C -1.64959236 -0.61693151 -1.65750894  C -0.02742036 0.99150649 -0.81767294  C 0.54214264 1.03042749 0.56286406  C -0.29820036 0.60133049 1.56843506  C -1.74924836 0.32069149 1.40917606  C 0.92986064 -1.48866151 1.86350906  C -1.45928836 -1.01928751 2.01288006  C -0.06221036 -0.65041651 2.32590406  C -1.80593636 -2.21341351 1.45339306  C 1.86107264 -1.11398351 0.77430306  C 0.97243564 -2.38871551 -1.20352394  1 2 1.0 4 1.0 14 1.5  2 13 2.0 24 1.0  3 13 1.0 16 1.0 23 1.5  4 5 2.0 24 1.0  5 6 1.0 9 1.0  6 7 2.0 22 1.0  7 8 1.0 19 1.0  8 23 1.0 24 2.0  9 10 1.0 22 1.0  10 11 1.0 14 1.0  11 12 1.0 18 1.5  12 14 1.0 15 2.0  13 15 1.0  14  15 16 1.0  16 17 2.0  17 18 1.0 21 1.0  18 20 1.0  19 21 2.0 23 1.0  20 21 1.0 22 2.0  21  22  23  24  C:/Users/SanCo/Desktop/C24.wfn |
| C24@Mydayis |
| %nprocshared=4  %chk=C:\Users\SanCo\Desktop\C24-Mydayis.chk  # opt wb97xd/6-31g(d) scrf=(cpcm,solvent=water) geom=connectivity out=wfn  Title Card Required  0 1  C -0.91827400 -2.08470900 0.00432100  C -0.11972800 -1.90688200 -1.23768700  C 0.58854200 0.26679500 -2.18158000  C 0.33465600 -2.10289300 0.80593000  C 0.49267800 -1.23218000 1.84997800  C 1.46167900 -0.10917700 1.74169800  C 2.19695400 0.05318600 0.59896600  C 2.02578800 -0.89329500 -0.53589100  C -0.58857300 -0.26676600 2.18177600  C -1.74229100 -0.24925200 1.44564300  C -2.02547700 0.89314400 0.53580600  C -2.19685200 -0.05321300 -0.59895000  C -0.38031800 -0.85599900 -2.07453700  C -1.91240200 -1.19499800 0.31043200  C -1.46178600 0.10921400 -1.74184700  C -0.49272500 1.23220100 -1.85004100  C -0.33468900 2.10285300 -0.80591300  C -1.13284000 1.92602000 0.43637800  C 1.91222700 1.19484700 -0.31041300  C 0.11969900 1.90683800 1.23767700  C 0.91827400 2.08473500 -0.00432300  C 0.38032600 0.85601700 2.07465800  C 1.74240100 0.24927400 -1.44573500  C 1.13272800 -1.92575900 -0.43634400  C -6.88075670 0.10513885 4.76445103  C -5.70749752 -0.01152270 4.01857946  C -4.76227666 -0.98125622 4.35234007  C -4.98957443 -1.83411723 5.43316401  C -6.16235837 -1.71711310 6.17910293  C -7.10821787 -0.74767914 5.84450243  H -7.62605850 0.86946403 4.50086976  H -5.52882072 0.66046810 3.16667291  H -4.24413031 -2.59860243 5.69616928  H -6.34178416 -2.38911307 7.03098124  H -8.03279360 -0.65575257 6.43258256  C -3.46758293 -1.11069145 3.52855936  H -3.14336398 -2.13038405 3.53157255  H -3.65251092 -0.79820185 2.52205457  C -2.37336461 -0.22228305 4.14905656  H -2.18031804 -0.54209532 5.15172955  C -2.84676338 1.24311047 4.15980007  H -3.45283834 1.42846207 3.29769919  H -3.41973694 1.42634753 5.04468735  H -1.99663462 1.89268444 4.14447833  H -0.31248916 0.24927323 3.76766513  H -0.77505474 -1.36281640 3.27749383  N -1.08359068 -0.33897801 3.31571058  1 2 1.0 4 1.0 14 2.0  2 13 2.0 24 1.0  3 13 1.0 16 1.0 23 2.0  4 5 2.0 24 1.0  5 6 1.0 9 1.0  6 7 2.0 22 1.0  7 8 1.0 19 1.0  8 23 1.0 24 2.0  9 10 2.0 22 1.0 47 1.0  10 11 1.0 14 1.0  11 12 1.0 18 2.0  12 14 1.0 15 2.0  13 15 1.0  14  15 16 1.0  16 17 2.0  17 18 1.0 21 1.0  18 20 1.0  19 21 2.0 23 1.0  20 21 1.0 22 2.0  21  22  23  24  25 26 1.5 30 1.5 31 1.0  26 27 1.5 32 1.0  27 28 1.5 36 1.0  28 29 1.5 33 1.0  29 30 1.5 34 1.0  30 35 1.0  31  32  33  34  35  36 37 1.0 38 1.0 39 1.0  37  38  39 40 1.0 41 1.0 47 1.0  40  41 42 1.0 43 1.0 44 1.0  42  43  44  45 47 1.0  46 47 1.0  47  C:/Users/SanCo/Desktop/C24-Mydayis.wfn |
| SiC23 |
| %nprocshared=4  %chk=C:\Users\SanCo\Desktop\SiC23.chk  # opt wb97xd/6-31g(d) scrf=(cpcm,solvent=water) geom=connectivity  Title Card Required  0 1  C 0.60428800 2.28288700 -0.00114300  C -0.41025800 1.89035000 -1.03165400  C -0.46762000 -0.56916300 -1.99895500  C -0.41012900 1.89155200 1.02993000  C -0.02166700 0.89929200 1.92707500  C -0.46746800 -0.56699400 1.99945600  C -1.41254800 -1.10355900 1.13672600  C 1.39832000 0.48194800 1.97793200  C 2.29899400 0.91188600 1.03622400  C 2.78135800 -0.02103600 -0.00005800  C 2.29886600 0.91079700 -1.03722500  C -0.02184000 0.89709600 -1.92787100  C 1.90041600 1.90576000 -0.00100800  C 1.39815100 0.47986300 -1.97845300  C 0.94185200 -0.91703500 -1.92786700  C 1.42866500 -1.84976400 -1.04101000  C 2.32965900 -1.33302400 0.00061500  C -0.84529700 -1.88122700 0.00096500  C 1.42868300 -1.84865400 1.04275900  C 0.48195500 -2.29852700 0.00116100  C 0.94192400 -0.91496700 1.92871900  C -1.41264500 -1.10487400 -1.13564000  C -1.41078900 1.63084400 -0.00075800  Si -2.67057400 0.04531000 0.00001600  1 2 1.0 4 1.0 13 2.0  2 12 1.5 23 1.0  3 12 1.0 15 1.0 22 1.5  4 5 1.5 23 1.0  5 6 1.0 8 1.0  6 7 1.5 21 1.0  7 18 1.0 24 1.0  8 9 2.0 21 1.0  9 10 1.0 13 1.0  10 11 1.0 17 1.5  11 13 1.0 14 2.0  12 14 1.0  13  14 15 1.0  15 16 2.0  16 17 1.0 20 1.0  17 19 1.0  18 20 1.5 22 1.0  19 20 1.0 21 2.0  20  21  22 24 1.0  23 24 1.0  24 |
| SiC23@Mydayis |
| %nprocshared=4  %chk=C:\Users\SanCo\Desktop\SiC23-Mydayis.chk  # opt wb97xd/6-31g(d) scrf=(cpcm,solvent=water) pop=nbo geom=connectivity  out=wfn  Title Card Required  0 1  C 1.90480500 -0.28254500 -2.25449600  C 3.30305200 0.11435400 -2.00732000  C 4.16705300 1.29495100 -0.02778800  C 2.16982600 -1.58283800 -1.51982000  C 1.36448700 -2.00099800 -0.48798000  C 1.92433500 -1.90414200 0.86608700  C 3.26449400 -1.57986600 1.10364600  C 4.10293100 -1.17587000 -0.03962100  C -0.02527000 0.22454000 -0.74888800  C 0.36753100 1.43819300 -0.04302000  C 1.24039500 1.86760600 -1.17096500  C 3.63396500 1.31549500 -1.40364800  C 0.89211000 0.57483000 -1.82368200  C 2.54421200 2.22403500 -0.95895300  C 3.09456200 2.20265500 0.42242000  C 2.27768600 1.80278100 1.46579800  C 0.86698000 1.42437900 1.25881800  C 3.57057500 -0.29886600 1.76502300  C 1.14355900 0.10761700 1.95895300  C 2.53386100 0.52314600 2.16683500  C 0.81340500 -1.09308000 1.37856400  C 4.43088700 0.10333500 0.62936100  C 3.56047600 -1.16757800 -1.31102800  C -5.95182000 1.80833300 0.36638600  C -4.68666800 1.30684800 0.07026100  C -4.53324400 0.02289600 -0.45835700  C -5.67451700 -0.75141500 -0.68531100  C -6.94108000 -0.25331400 -0.39189700  C -7.08303500 1.02892400 0.13525400  H -6.05390600 2.80892000 0.77598300  H -3.80579300 1.91819100 0.25051000  H -5.56926400 -1.74968100 -1.10399700  H -7.81864200 -0.86483300 -0.57994400  H -8.07055300 1.42035100 0.36025000  C -3.15955200 -0.52869700 -0.75742700  H -3.18848500 -1.09417000 -1.69702000  H -2.44908500 0.29211400 -0.89706900  C -2.65323700 -1.45025400 0.36166200  H -3.36297400 -2.27644500 0.47368500  C -2.48268900 -0.74964100 1.70131300  H -1.85119300 0.13939100 1.60922100  H -3.46222700 -0.43589000 2.06982000  H -2.03486800 -1.41721700 2.44298000  H -1.19290400 -2.90413500 0.53312700  H -1.47257500 -2.46879900 -1.00168100  N -1.35276000 -2.08779000 -0.06074200  Si -0.01573500 -1.22963400 -0.03435400  1 2 1.0 4 1.0 13 1.5  2 12 2.0 23 1.0  3 12 1.0 15 1.0 22 1.5  4 5 2.0 23 1.0  5 6 1.0 47 1.0  6 7 1.5 21 1.0  7 8 1.0 18 1.0  8 22 1.0 23 2.0  9 10 1.0 13 1.0 47 1.0  10 11 1.0 17 1.5  11 13 1.0 14 2.0  12 14 1.0  13  14 15 1.0  15 16 2.0  16 17 1.0 20 1.0  17 19 1.0  18 20 2.0 22 1.0  19 20 1.0 21 2.0  20  21 47 1.0  22  23  24 25 1.5 29 1.5 30 1.0  25 26 1.5 31 1.0  26 27 1.5 35 1.0  27 28 1.5 32 1.0  28 29 1.5 33 1.0  29 34 1.0  30  31  32  33  34  35 36 1.0 37 1.0 38 1.0  36  37  38 39 1.0 40 1.0 46 1.0  39  40 41 1.0 42 1.0 43 1.0  41  42  43  44 46 1.0  45 46 1.0  46 47 1.0  47  C:\Users\SanCo\Desktop\SiC23-Mydayis.wfn |

Table S2 reports the raw values of the adsorption energy, Gibbs free energy, enthalpy, and the BSSE correction for each of the structures designed in this work. Providing these data ensures the repeatability of all calculated values and enables independent verification of the computational results presented in this study.

| Table S2. DFT-computed total energy, Gibbs free energy, enthalpy, and BSSE values for Mydayis and the designed C_24_-based nanostructures, along with their optimized adsorption complexes. | | | | |
| --- | --- | --- | --- | --- |
| Structure | **Energy (Hartree)** | **Gibbs Free Energy (Hartree)** | **Enthalpy (Hartree)** | **BSSE**  **(Hartree)** |
| Mydayis | -405.415 | -405.236790 | -405.191289 | --------- |
| C_24_ | -913.504 | -913.396636 | -913.352260 | --------- |
| BC_23_ | -900.283 | -900.180160 | -900.134855 | --------- |
| SiC_23_ | -1164.893 | -1164.790471 | -1164.744312 | --------- |
| C_24_@Mydayis | -1318.966 | -1318.637418 | -1318.569755 | 0.008945 |
| BC_23_@Mydayis | -1305.790 | -1305.474935 | -1305.405149 | 0.007380 |
| SiC_23_@Mydayis | -1570.402 | -1570.075355 | -1570.004345 | 0.008000 |
